# Ehf and Fezf2 regulate late medullary thymic epithelial cell and thymic tuft cell development

**DOI:** 10.3389/fimmu.2023.1277365

**Published:** 2024-02-14

**Authors:** Sören Lammers, Victor Barrera, Philip Brennecke, Corey Miller, Joon Yoon, Jared Balolong, Mark S. Anderson, Shannan Ho Sui, Lars M. Steinmetz, Ulrich H. von Andrian, Kristin Rattay

**Affiliations:** ^1^Institute for Theoretical Physics, Heidelberg University, Heidelberg, Germany; ^2^Bioinformatics Core, Harvard T.H. Chan School of Public Health, Boston, MA, United States; ^3^Department of Genetics, Stanford University, School of Medicine, Stanford, CA, United States; ^4^Stanford Genome Technology Center, Stanford University, Stanford, CA, United States; ^5^Diabetes Center, University of California, San Francisco (UCSF), San Francisco, CA, United States; ^6^Genome Biology Unit, European Molecular Biology Laboratory (EMBL), Heidelberg, Germany; ^7^Department of Immunology & HMS Center for Immune Imaging, Harvard Medical School, Boston, MA, United States; ^8^The Ragon Institute of MGH, MIT and Harvard, Cambridge, MA, United States; ^9^Pharmacological Institute, Biochemical Pharmacological Center, University of Marburg, Marburg, Germany

**Keywords:** thymus, central tolerance, medullary thymic epithelial cell, Tuft cells, Fezf2, Ehf

## Abstract

Thymic epithelial cells are indispensable for T cell maturation and selection and the induction of central immune tolerance. The self-peptide repertoire expressed by medullary thymic epithelial cells is in part regulated by the transcriptional regulator Aire (Autoimmune regulator) and the transcription factor Fezf2. Due to the high complexity of mTEC maturation stages (i.e., post-Aire, Krt10+ mTECs, and Dclk1+ Tuft mTECs) and the heterogeneity in their gene expression profiles (i.e., mosaic expression patterns), it has been challenging to identify the additional factors complementing the transcriptional regulation. We aimed to identify the transcriptional regulators involved in the regulation of mTEC development and self-peptide expression in an unbiased and genome-wide manner. We used ATAC footprinting analysis as an indirect approach to identify transcription factors involved in the gene expression regulation in mTECs, which we validated by ChIP sequencing. This study identifies Fezf2 as a regulator of the recently described thymic Tuft cells (i.e., Tuft mTECs). Furthermore, we identify that transcriptional regulators of the ELF, ESE, ERF, and PEA3 subfamily of the ETS transcription factor family and members of the Krüppel-like family of transcription factors play a role in the transcriptional regulation of genes involved in late mTEC development and promiscuous gene expression.

## 1 Introduction

Immunological tolerance is essential in order to avoid immune reactions toward self-peptides, namely, autoimmune reactions. Central tolerance induction occurs in the thymus mediated by different sets of thymic antigen-presenting cells (APCs), including thymic epithelial cells (i.e., cortical thymic epithelial cells (cTECs) and medullary thymic epithelial cells (mTECs)), dendritic cells (DCs), and thymic B cells (1–5). These thymic APCs present endogenously transcribed and imported peripheral peptides by major histocompatibility complex (MHC) class I and II molecules on their surfaces to develop T cells. When clonotypic TCRs bind to self-antigen/MHC complexes above a certain threshold, the respective auto-reactive T cells will be either purged from the repertoire by deletion or fate-diverted into regulatory T cells. Among these thymic APCs, mTECs stand out due to their ability to promiscuously express the majority of tissue-restricted self-antigens (TRAs) and, thus, by themselves, largely procure self-tolerance against peripheral tissues (6, 7).

One of the main features of promiscuous gene expression (pGE) is the mosaic expression pattern by which each TRA is expressed in approximately 1-5% of the mTECs at a certain point in time. This characteristic is conserved between mice, rats, and humans (8–13) Yet, this substantial heterogeneity at the single cell (SC) level faithfully adds up to the complete repertoire of self-antigens at the population level (7, 14–17). Due to the pronounced heterogeneity in pGE, the cellular and, in particular, the molecular regulation underneath remains challenging to reveal (18–23). In the past, several studies focused on analyzing the nature of TRA mosaic expression patterns addressing the question of whether this phenomenon is based on stochastic or regulated processes. Early studies performed on bulk mTEC populations were unable to observe predictable recurrent gene expression patterns (10, 13), as, due to high cell-to-cell variability in gene expression, the patterns of subpopulations were not detectable on the complete population level at the time. However, recent studies using single-cell technology and selective enrichment for TRA-expressing mTEC subsets reported recurring gene expression patterns in mTECs in mice and humans (7, 11, 12, 14, 16). The factors involved in the regulation of those recurring gene expression patterns, which give rise to the characteristic mosaic expression in mTEC, remain to be identified. While the transcriptional regulator Autoimmune regulator (Aire) has been shown to be responsible for targeting part of the self-antigen gene pool (i.e., 49%; 533 Aire-dependent and 3260 Aire-enhanced TRAs of a total of 7740 detected TRA genes so far (6)), other factors and mechanisms acting in concert or independently of Aire have to be involved to account for the comprehensive tolerance coverage afforded by pGE. The work of Takayanagi and colleagues identified Fezf2 as a transcription factor involved in the regulation of some Aire-dependent but mostly Aire-independent TRA gene expression in mTECs (21, 22). The promiscuously expressed genes in mTECs comprise a diverse range of biological functions and tissue origins and vary greatly in their regulatory elements and promoter regions. Remarkably, in the context of pGE, peripheral tissue-specific transcription factors were shown to be dispensable for the respective thymic gene expression (13, 24, 25).

Medullary thymic epithelial cells can be distinguished into different developmental stages based on the maturation markers MHCII, Aire, Keratin 10 (Krt10), and Involucrin (Ivl) as early mTECs (MHCII^low^Aire^neg^Krt10^neg^Ivl^neg^), mature Aire^pos^-mTECs (MHCII^high^Aire^pos^Krt10^neg^Ivl^neg^), mature Aire^neg^-mTECs (MHCII^high^Aire^neg^Krt10^neg^Ivl^neg^), and late mTECs (MHCII^low^Aire^neg^Krt10^pos^Ivl^pos^). The early developmental stage includes podoplanin-expressing (Pdpn^+^) junctional thymic epithelial cells (jTECs) (17, 26). The late mTEC stage comprises post-Aire cornified (Krt10^+^) mTECs (27, 28) as well as Tuft-mTECs (Dclk1^+^) (15, 20, 29, 30), microfold mTECs (Gp2^+^) and other recently described mimetic mTECs (31). The transcriptional regulation during mTEC development is insufficiently understood to date. In order to reveal the supposedly complex network of transcriptional regulators necessary to accomplish the thymic expression of self-antigens, we aimed to apply a genome-wide screening method. Additionally, we analyzed the role of Fezf2 in the regulation of newly identified transcription factors and mTEC development. The usage of footprinting analysis on ATAC sequencing allows for unbiased, genome-wide profiling of a broad and diverse set of transcription factors (TF) in which the sequencing footprints of bound transcription factor binding sites (TFBS) in the promoter regions of expressed genes served as an indirect readout for TF binding (32, 33). This approach led to the identification of multiple transcription factors of the ELF, ESE, and PEA3 subfamily of the ETS transcription factor family and members of the Krüppel-like family of transcription factors to be involved in late mTEC development and the regulation of promiscuous gene expression. Using ChIPmentation, we validated the binding of the transcription factors Ehf, Elf3, Klf4, and Fezf2 to the promoter regions of mTEC gene signatures (34, 35). We show that Fezf2 and Ehf are involved in the regulation of late developmental gene signatures implicated in cornification and keratinization in mTECs and that Fezf2 regulates Tuft-mTEC-specific gene signatures. Moreover, using conditional knockout mice (FoxN1-cre/Fezf2-flox), we identify Fezf2 to be essential for the development of thymic Tuft-mTECs (15, 20, 29, 30). This newly identified role of Fezf2 on the regulation of late mTEC and Tuft-mTEC subsets contributes to deciphering the high cellular complexity of the thymic epithelial cell landscape.

## 2 Results

### 2.1 Division of gene expression patterns reveals enrichment for distinct TFBMs in promoter regions of mTEC subsets

**Figure 1:**
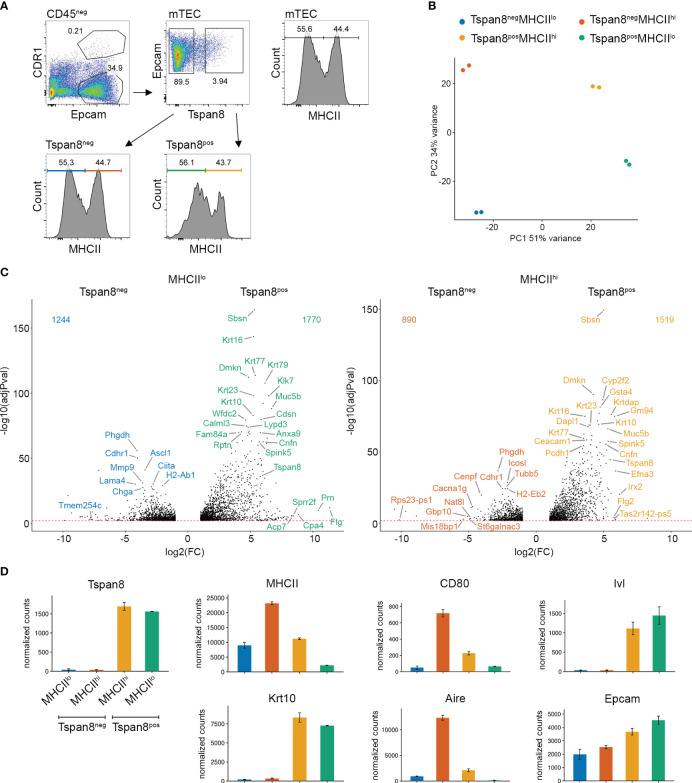
Co-expression groups in MHCII low and high Tspan8^pos^ mTEC populations analyzed by RNA sequencing. **(A)** FACS gating strategy for Tspan8^pos^ mTEC isolation. Pregated on life-singlets-CD45^neg^, mTECs are isolated as Epcam^pos^CDR1^neg^. **(B)** Principal component analysis of Tspan8^neg^MHCII^lo^ (blue), Tspan8^neg^MHCII^hi^ (red), Tspan8^pos^MHCII^lo^ (green), and Tspan8^pos^MHCII^hi^ (orange) mTECs using the top 1000 variable genes from the RNA sequencing. **(C)** Volcano plots of differential gene expression between Tspan8^neg^MHCII^lo^ (blue) compared to Tspan8^pos^MHCII^lo^ (green) left panel and Tspan8^neg^MHCII^hi^ (red) compared to Tspan8^pos^MHCII^hi^ (orange) right panel. **(D)** Normalized gene expression counts for Tspan8, MHCII, CD80, Ivl, Krt10, Aire, and Epcam in Tspan8^neg^MHCII^lo^ (blue), Tspan8^neg^MHCII^hi^ (red), Tspan8^pos^MHCII^hi^ (orange) and Tspan8^pos^MHCII^lo^ (green) mTEC populations. Normalized mean counts ± SEM. See also **Supplementary Figure 1**.

The complexity of the mTEC population necessitates a disentanglement of the heterogeneous subpopulations to a degree that gene expression patterns are detectable and the mechanistic regulation underlying the mosaic expression is discernable. This can be accomplished either by single-cell RNA-seq analysis, which to date still has limitations in sequencing depth for low transcribed genes, or by isolating specific subsets of mTECs, which are small enough, to reduce the heterogeneity sufficiently. The distinction between immature and mature mTECs (MHCII^lo^ and MHCII^hi^) has been used in the past to segregate mTEC subsets. However, this proved to be an insufficient degree of separation in order to resolve gene expression patterns and the underlying regulatory mechanisms, not only due to the complexity of TRA expression but also because of the late mTEC stages (post-Aire mTECs and Tuft-mTECs) that downregulate MHCII and fall into the MHCII^lo^ subset together with the immature mTEC stage. We previously identified the markers Gp2, Pdpn, and Tspan8 for the isolation of distinct developmental stages of mTECs (12), which were further characterized as podoplanin-expressing (Pdpn^+^) junctional thymic epithelial cells (jTECs) (17, 26), and Gp2-expressing (Gp2^+^) microfold mTECs (31). FACS-based enrichment for exemplary TRA-specific mTECs was shown to be a reliable method in order to purify mTEC subsets representing 1-5% of the mTEC population (depending on the TRA) (11, 12, 14). Among such TRA-positive mTECs, co-expression patterns could be revealed and were shown to be evolutionarily conserved between species. We used Tspan8 (Tetraspanin-8) as an exemplary TRA that is expressed in mature MHCII^hi^ mTECs and late post-Aire MHCII^lo^ mTECs (**Figure 1A**, **Supplementary Figure 1A**) (12, 14). Tspan8-positive mTECs (Tspan8^pos^) account for approximately 4% of mTECs, of which 56% fall into the MHCII^lo^ and 44% into the MHCII^hi^ subset of mTECs. This allowed us to address the occurrence of gene expression and regulation patterns in a TRA-pos mTEC subset (Tspan8^pos^ compared to Tspan8^neg^) while also addressing potential maturation-dependent effects (MHCII^lo^ compared to MHCII^hi^). Therefore, we FACS-isolated four distinct mTEC subpopulations, namely, Tspan8^pos^MHCII^lo^, Tspan8^pos^MHCII^hi^, Tspan8^neg^MHCII^lo^, and Tspan8^neg^MHCII^hi^ and performed RNA sequencing (RNA-seq) on them (**Figure 1B**). The identified differentially expressed genes (**Figure 1C**, **Supplementary Figure 1A**) have miscellaneous biological functions and tissue origin, a previously described feature of promiscuously expressed genes (12, 19, 36). As a quality check, we analyzed the Tspan8 and MHCII mRNA expression levels in our RNA sequencing dataset (**Figure 1D**). The mRNA expression levels of Tspan8 and MHCII in the four mTEC subsets correlated well with the protein expression levels used for FACS sorting. We additionally validated those expression levels using quantitative real-time PCR (**Supplementary Figure 1B**). Based on the mTEC maturation markers CD80, Ivl, Krt10, Aire, and Epcam, the four mTEC subsets were analyzed with respect to their developmental stage (37–40). The isolated mTEC subsets are characterized by differences in the expression of early-to-late mTEC developmental signatures, giving rise to a putative developmental sequence of Tspan8^neg^MHCII^low^ (containing immature mTECs) – Tspan8^neg^MHCII^high^ (mature mTECs) - Tspan8^pos^MHCII^high^ (mature mTEC subset) – Tspan8^pos^MHCII^low^ (late mTECs/post-Aire mTECs). At first, MHCII and CD80 are lowly expressed (immature mTECs), followed by an upregulation of MHCII, CD80, and Aire (mature mTECs), followed by an additional increase in Ivl and Krt10 expression, markers of keratinization and cornification (Tspan8 mature mTEC subset) in which MHCII, CD80, and Aire start to be downregulated, and finally, a stage with the lowest MHCII, CD80, and Aire expression levels in combination with high expression levels for Krt10 and Ivl (late mTECs/post-Aire mTECs). Notably, we observed Tuft-signature genes (Dclk1, Avil, Trpm5, Alox5, Plcb2, and different taste 2 receptors (Tas2r)) among the most differentially expressed genes in the MHCII^low^ subsets, independent of the Tspan8 expression level (in Tspan8^neg^MHCII^low^ and Tspan8^pos^MHCII^low^) (**Supplementary Figure 1A**). Thus, the Tspan8^neg^MHCII^low^ mTEC population is a mixture of MHCII^low^ immature mTECs and mimetic mTECs. The recently identified and characterized Tuft-mTECs are described to reflect a late mTEC developmental stage contributing to central tolerance induction and iNKT-cell development (15, 20, 27).

It has been previously described that co-expressed genes in mTECs exhibit distinct regulatory motifs in their upstream promoter regions (12, 17). The enrichment of a binding motif in promoter regions of co-expressed genes serves as an indication for a putative role in the transcriptional regulation; however, whether those transcription factors actually bind those regions and regulate transcription in these specific mTEC subsets needed to be addressed. In an indirect, genome-wide, and unbiased approach, we applied ATAC sequencing and ATAC footprinting analysis on the four mTEC subsets to identify open genomic regions bound by transcription factors, which are putatively involved in the regulation of promiscuous gene expression in the mTEC subsets.

### 2.2 Regulatory motif occupancy adverts the regulatory network underlying promiscuous gene expression and mTEC maturation

The transcription factors complementing the transcriptional regulation by Aire and Fezf2 in mTECs remained to be identified. The transcription factors known to regulate gene expression in peripheral tissues were shown to be dispensable for the respective gene regulation in the thymus. Therefore, in order to identify the transcription factors involved in the regulation of self-peptide and maturation-dependent gene expression in thymic stroma cells, it was important to utilize a comprehensive and unbiased approach. ATAC footprinting analysis allows for an indirect readout of occupied TFBMs in promoter regions. Through this approach, the list of putative TFs identified based on the enrichment of TFBM enrichments in the promoter regions can be further narrowed down to TFs for which putative binding is detected.

ATAC-seq footprinting analysis is sensitive to mitochondrial DNA overrepresentation in sequencing data sets. Yet, a sufficient sequencing depth on the chromosomes and genes to be analyzed is essential for robust identification of binding events. Therefore, at first, we analyzed the chromosomal distribution and sequencing fragment length of our ATAC-seq datasets. The representation of mitochondrial DNA reads was relatively low in all four mTEC subsets and all replicates (**Supplementary Figure 2A**). Furthermore, the high proportion of reads in fragment length of nucleosome-free regions, being below 100 bps as opposed to mono-, di-, or tri-nucleosome bound regions, provided ideal conditions for the ATAC footprinting analysis (**Supplementary Figure 2B**).

**Figure 2:**
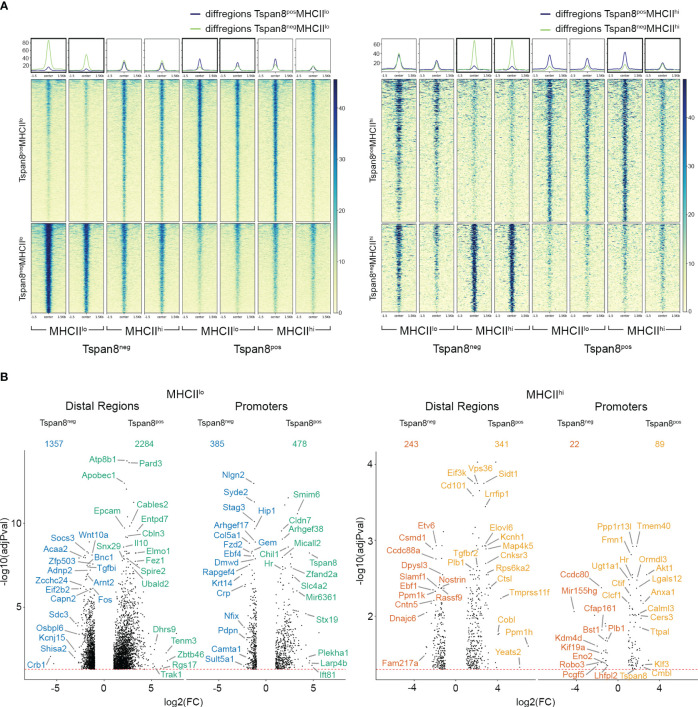
Differential ATAC-seq reads in Tspan8^pos^ compared to Tspan8^neg^ mTEC subpopulations. **(A)** Differential ATAC reads in Tspan8^pos^MHCII^lo^ compared to Tspan8^neg^MHCII^lo^ on the left and Tspan8^pos^MHCII^hi^ compared to Tspan8^neg^MHCII^hi^ on the right. Heatmaps show normalized reads within +/- 1.5 kb around the differential regions. **(B)** Volcano plot showing differential ATAC reads between Tspan8^neg^MHCII^lo^ (blue) compared to Tspan8^pos^MHCII^lo^ (green), left panel and Tspan8^neg^MHCII^hi^ (red) compared to Tspan8^pos^MHCII^hi^ (orange), right panel. The nearest genes to the differential distal regions and promoter regions are depicted. Numbers indicate the total amount of genes identified for each classification. See also **Supplementary Figures 2**, **3**.

We analyzed the ATAC reads of mTECs Tspan8^pos^MHCII^lo^, Tspan8^pos^MHCII^hi^, Tspan8^neg^MHCII^lo^, and Tspan8^neg^MHCII^hi^ (**Figure 2A**, **Supplementary Figures 2C**, **3A**). The differential ATAC reads in distal regions and promoter regions for Tspan8^pos^MHCII^lo^ compared to Tspan8^neg^MHCII^lo^ (Tspan8 pos/neg in MHCII^low^-mTECs) and Tspan8^pos^MHCII^hi^ compared to Tspan8^neg^MHCII^hi^ (Tspan8 pos/neg in MHCII^high^-mTECs) were assigned to their nearest gene (**Figure 2B**). This comparison was used to analyze TRA-specific regulatory patterns. Additionally, we compared Tspan8^neg^MHCII^hi^ to Tspan8^neg^MHCII^lo^ (MHCII high/low of the Tspan8^neg^-mTEC subsets) and Tspan8^pos^MHCII^hi^ to Tspan8^pos^MHCII^lo^ (MHCII high/low of the Tspan8^pos^-mTEC subsets) to analyze regulatory patterns involved in the transcriptional control of maturation processes (**Supplementary Figure 3B**). We observed distinct ATAC reads in the MHCII^lo^ and MHCII^hi^ mTECs (**Supplementary Figures 3A, B**) and also specific ATAC read signatures for the Tspan8^neg^ and Tspan8^pos^ mTECs (**Figures 2A, B**), respectively. Noteworthy, the Tspan8 gene promoter was among the gene promoters with ATAC reads in the Tspan8^pos^ FACS isolated mTEC population, serving as a positive control, showing that Tspan8 gene expression correlated with open chromatin at the Tspan8 promoter region. Next, we correlated the ATAC-seq reads in promoter regions with the level of gene expression in the RNA-seq dataset. As expected, we observed a correlation of open chromatin regions, represented by the ATAC-seq reads, in the promoter region of expressed genes in the mTEC datasets (**Supplementary Figures 4A, B**).

### 2.3 ATAC footprinting analysis indicates a role of the transcription factors Elf3, Elf5, Ehf, Klf1, and Klf4 in the transcriptional control of gene expression in mTECs

**Figure 3:**
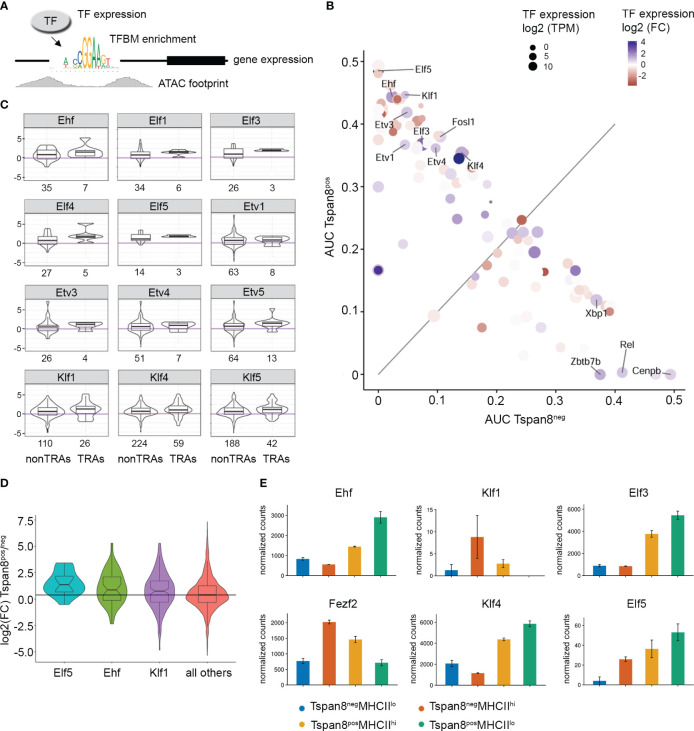
Identification of candidate transcription factors responsible for the regulation of gene expression in Tspan8^pos^ mTECs by correlation of differential ATAC-seq footprinting with the corresponding transcription factor and target gene expression. **(A)** Schematic representation of the experimental setup. A combinatorial enrichment analysis (Tspan8^pos^ vs. Tspan8^neg^) was performed for transcription factor expression with corresponding enrichment of the specific TFBM in the promoter region, ATAC footprint signal, and target gene expression. **(B)** Area under the curve (AUC) values of differential footprint enrichment in TRA gene promoters are plotted for Tspan8^pos^ MHCII^lo^ compared to Tspan8^neg^ MHCII^lo^ mTECs. Dot size shows the transcription factor (TF) expression in log2 transcripts per million (TPM). Color code indicates the log2 fold change of TF expression between Tspan8^pos^ and Tspan8^neg^. **(C)** Expression of nearest genes (log2 fold change) to differential ATAC peak with respective TF footprint for Tspan8^pos^ compared to Tspan8^neg^ mTECs. Numbers indicate the total number of target genes of the corresponding TF for non-TRAs (left bar) and TRAs (right bar) each. **(D)** Expression of nearest genes to differential ATAC peak with respective TF footprint; shown is the log2 fold change of gene expression (Tspan8^pos^ vs. Tspan8^neg^) for the transcription factors Elf5, Ehf, and Klf1. **(E)** Normalized gene expression counts for Ehf, Fezf2, Klf1, Klf4, Elf3, and Elf5 in Tspan8^neg^MHCII^lo^ (blue), Tspan8^neg^MHCII^hi^ (red), Tspan8^pos^MHCII^hi^ (orange) and Tspan8^pos^MHCII^lo^ (green) mTEC populations. Normalized mean counts ± SEM. See also **Supplementary Figure 4**.

We performed footprinting analysis on our ATAC-seq data and explored the ATAC footprints in promoter regions of co-expressed genes (Tspan8^pos^ compared to Tspan8^neg^ mTECs) to identify the transcription factors regulating gene expression in the isolated mTEC subsets. To this end, our underlying derivation was that for those transcription factors that are involved in the transcriptional regulation of gene expression, the following criteria would apply: **I.** the target gene is expressed and detectable on the mRNA level, **II.** the presence of the TFBM in the promoter region of the expressed gene, **III.** ATAC-seq footprint around the TFBM in the promoter region of expressed genes serves as an indirect read-out for an occupied TFBM in an open chromatin region, **IV.** the corresponding transcription factor predicted to be binding to the TFBM in the promoter region is expressed in our mTEC subset (**Figure 3A**).

Thus, we used the differential gene expression analysis of our RNA-seq data and identified the enriched TFBMs in the promoter region of the differentially expressed genes. We then correlated those enriched TFBMs in promoter regions of differentially expressed genes with our ATAC footprinting analysis. The observed ATAC footprints in the promoter region of differentially expressed genes served as an indirect detection of transcription factor binding to the promoter region because the TF binding would make that particular region inaccessible to the Tn5 transposase in the ATAC assay, resulting in a defined small region devoid of reads within a larger region of open chromatin with corresponding ATAC reads. Based on this indirect binding identification, we then analyzed the expression intensity of the predicted corresponding transcription factors that would bind the respective TFBMs (**Figure 3B**). Through this indirect genome-wide screening approach, we identified transcription factors of the ELF, ESE, ERF, and PEA3 subfamily of the ETS transcription factor family, and members of the Krüppel-like family of transcription factors to be involved in gene expression regulation in mTECs (**Figures 3B, C**). The expression levels of the nearest gene to the differential ATAC footprint were identified and the differential gene expression of the target genes, in particular, TRA genes, was plotted for those transcription factors that were identified in the screening (**Figure 3C**). Elf5, Ehf, and Klf1 were the top candidates identified by our approach (**Figures 3B, D**) based on the differential footprint enrichment in TRA gene promoters, followed by Elf3 and Klf4, which also showed enriched footprints, but slightly lower enrichment values. However, Ehf, Elf3, and Klf4 showed higher TF expression levels (dot size, **Figure 3B**) and fold change in expression (dot color scale) compared to Klf1 and Elf5. The mRNA expression levels of the identified transcription factors analyzed by RNA-seq and qRT-PCR are plotted in (**Figure 3E**, **Supplementary Figure 4D**) and compared to the expression levels of Fezf2. Hereby, it became apparent that the identified candidate TFs varied substantially in their expression levels, and even though Klf1 and Elf5 were differentially expressed and showed enrichment for the ATAC footprint, the isolated mTEC subsets did not show high expression levels for these TFs. This could be due to the subset selection that we looked at, meaning that, at the particular time when we isolated this subset, the mRNA for Klf1 might not have been transcribed at the maximum level yet and might have been upregulated in a later, subsequent developmental stage, or it may not be transcribed at the maximum level anymore, having been downregulated again. Whereas Ehf, Elf3, and Klf4 showed high expression levels and strong enrichment in Tspan8^pos^ mTECs, with the highest expression in the Tspan8^pos^MHCII^low^ mTEC subset. Based on the expression marker analysis of the subsets (**Figure 1D**), these data suggest that those transcription factors play a role in gene regulation in late and post-Aire mTECs. Next, we used ChIP-sequencing to test whether the predicted transcription factors were in fact binding to the promoter regions of the co-expressed genes in mTECs.

### 2.4 Ehf, Elf3, Klf4, and Fezf2 are binding to the promoter regions of genes associated with late mTEC development

We used an ultrasound-based nuclei extraction method (Nexson: Nuclei Extraction by Sonnification) (41) followed by ChIPmentation sequencing to analyze the binding sites of the identified transcription factors (34, 35). In ChIPmentation, as opposed to classical ChIP-seq protocols, the chromatin immunoprecipitation and tagmentation are combined and washing steps are reduced. The tagmentation by the Tn5 transposase is performed directly on bead-bound chromatin. This method is particularly well suited to perform ChIP-seq experiments on low input samples. We performed ChIPmentation-seq on mTECs for the TFs Ehf, Elf3, Fezf2, and Klf4. We included Fezf2 in our analysis to be able to compare the transcriptional profiles that we obtained for the other TFs to a TF already known and described to regulate gene expression in mTECs. We also tried to establish the ChIPmentation protocol for Klf1 and Elf5 but could not obtain sufficient amounts of chromatin after IP to perform ChIPmentation-seq on mTECs for these TFs. Likely, the transcription factors were expressed at too low levels (**Figure 3E**), leading to insufficient amounts of precipitated chromatin. Hence, we could not analyze the transcription factor binding of Klf1 and Elf5 in mTECs using ChIP-seq to test the prediction from the ATAC footprinting analysis.

**Figure 4:**
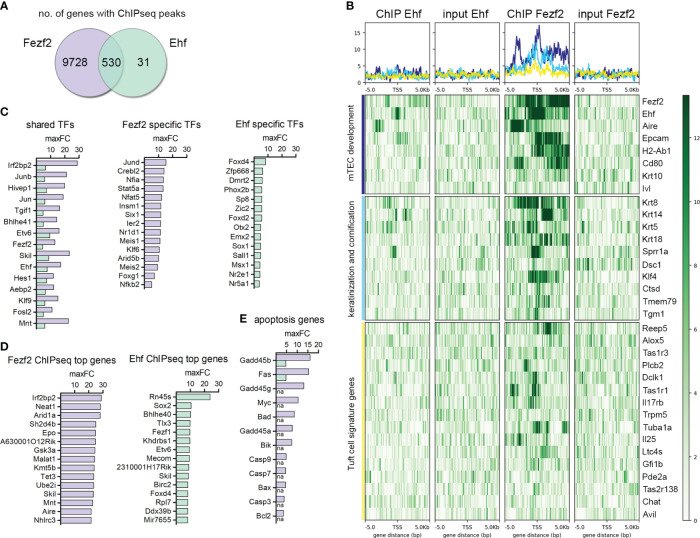
ChIP-seq for Fezf2 and Ehf on mTECs identifies regulation of late mTEC development-associated gene expression and Tuft cell signatures. **(A)** Venn-diagram comparison of genes with a Fezf2 and/or Ehf binding site within +/- 5000 bp from their TSS in mTECs. **(B)** ChIP-seq signal distribution within +/- 5000 bp from the TSS of mTEC developmental marker genes, genes with annotated functions in keratinization, cornification, and Tuft cell signature genes. From left to right, the four panels indicate the peak distribution in the Ehf ChIP, Ehf input control, Fezf2 ChIP, and Fezf2 input control. Y-axis and color scale represent the number of reads per 50bp bin. **(C)** Transcription factors with the highest maxFC enrichment in the ChIP compared to the input control targeted by Fezf2 and Ehf (shared TFs, left panel), targeted specifically by Fezf2 (middle panel), or targeted specifically by Ehf (right panel). For each gene, maxFC represents the maximum value for the signal enrichment among all peaks within +/- 5000bp from their TSS. **(D)** Fezf2 (left) and Ehf (right) target genes with the highest maxFC enrichment in the ChIP compared to the input control. **(E)** Apoptosis and DNA damage-related genes targeted by Fezf2 and/or Ehf. Indicated is the maxFC enrichment of the respective ChIP compared to its input control. See also **Supplementary Figure 5**.

Due to the high complexity of the mTEC population, which is constructed by the developmentally distinct subsets and diverse TRA-specific subsets, we expected the transcription factors responsible for the regulation of self-peptide expression in mTECs to be restricted in a subset-specific manner as well. Assuming a multi-factor transcriptional network of factors that regulates the co-expression patterns during mTEC development in consecutive order, we expected each individual TF to be regulating specific target gene groups at particular developmental time points and TFs to be acting in concert. In total, 561 genes mapped to Ehf ChIP-seq peaks, with reads located +/- 5000 bp of the TSS in comparison to input control (**Figure 4A**), whereas the Fezf2 ChIP-seq peaks mapped to 10.258 genes in total. Surprisingly, 94.5% of the genes identified in the Ehf ChIP-seq were also targeted by Fezf2, whereas only 5.2% of the Fezf2-targeted genes also showed binding of the transcription factor Ehf. Previously described Fezf2-dependent genes such as Kctd15, Asxl3, Prokr2, Ckmt1, Kif26a, Krt10, and others were analyzed for their peak intensity in our Fezf2 ChIPmentation sequencing dataset (**Supplementary Figure 5A**). We observed direct binding of Fezf2 to the regions +/- 5000bp from the TSS of the listed Fezf2-dependent genes. Other previously described Fezf2-dependent genes such as Ttr, Apoc3, Csmp3, Klk1b16, Smtnl1, Cd177, and Pck1 did not show enriched peaks in the ChIP compared to the input control in our analysis. It is possible that those genes could be influenced indirectly by Fezf2, regulated by different transcription factors, which themselves are Fezf2-dependently expressed.

**Figure 5:**
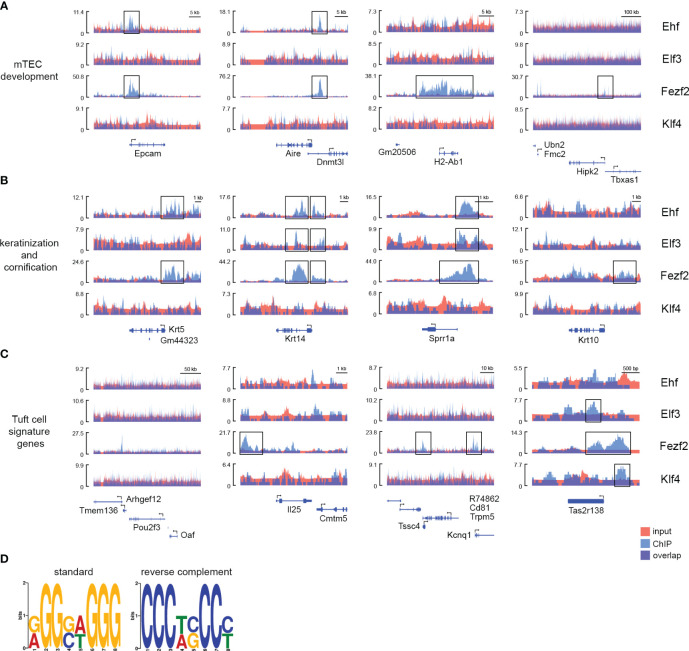
ChIP-seq peaks for Fezf2, Ehf, Elf3, and Klf4 on mTECs overlap in the promoter region of late mTEC development-associated genes and Tuft cell signature genes. Signal density plots indicating the read density around the TSS **(A)** of mTEC maturation and marker genes Epcam, Aire, H2-Ab1, and Hipk1, **(B)** of keratinization and cornification markers Krt5, Krt14, Sprr1a, and Krt10, **(C)** of the Tuft cell signature genes Pou2f3, Il25, Trpm5, and Tas2r138. ChIP (blue) and input control (red) tracks for the transcription factors Ehf, Elf3, Fezf2, and Klf4 are shown. The Y-axis represents the λ score from MACS2, i.e., Read length (nt) * Total read number/Effective genome length (nt). **(D)** Logo of the predicted consensus sequence for the Fezf2 TFBM in mice based on our Fezf2 ChIP-seq experiment and a Stamp-based species comparison to the Fezf2 TFBM in zebrafish published by Chen et al. The predicted Fezf2 binding motif found in MEME (E-value 5.5e-008, 3456 associated sites) was statistically similar to the MEME motif from the supplementary file in Chen et al., according to STAMP (E-value 2.0902e-02).

We detected 27 genes with Elf3 ChIP peaks and 40 genes with Klf4 ChIP peaks +/- 5000bp from the TSS. These numbers were much lower compared to the number of genes with Ehf ChIP peaks. The interpretation of so few hits has to be done with caution. We observed few genes among those hits with peaks at genes that we also observed to be targeted by Ehf or Fezf2. We detected Elf3 to bind to Krt14 and Sprr1a genes involved in keratinization and cornification during mTEC development. Klf4 and Elf3 binding was detected at the Taste receptor family member Tas2r138, which is expressed in Tuft-mTECs (**Figure 5**). We further analyzed the Fezf2 and Ehf target genes in order to better understand the nature of their high overlap in their gene targets and their role in gene expression regulation in mTECs. We performed gene ontology enrichment analysis, identified enriched functional classes, and compared the top hits (maxFC) of Fezf2 and Ehf target genes (**Figures 4B–E**, **Supplementary Figure 5C**). Enriched among the target genes were mTEC developmental marker genes such as Aire and Epcam in the Fezf2 and Ehf ChIP-seq, whereas the maturation markers H2-Ab1 (MHCII) and CD80 and late mTEC developmental marker genes Krt10 and Ivl were bound by Fezf2 only. Furthermore, mTEC development-associated genes involved in keratinization and cornification, such as Krt8, Krt14, Krt5, Sprr1a, and Dsc1, showed peaks located +/- 5000 bp of the TSS in the Fezf2 and Ehf ChIP-seq and Krt18, Klf4, Ctsd, Tmem79, and Tgm1 showed peaks in the Fezf2 ChIP-seq only (**Figures 4B**, **5B**). Next, we wondered whether broader functioning transcriptional regulators like Fezf2 and Ehf might act on the regulation of further TFs, thereby initiating a downstream diversification and sub-setting of the gene regulation, leading to mosaic expression patterns in mTECs.

For this purpose, we identified the TFs targeted by Fezf2 and Ehf (shared TFs), those targeted by Fezf2 only (Fezf2 specific TFs), and those with peaks specifically identified in the Ehf ChIP-seq (Ehf specific TFs) (**Figure 4C**). Among the enriched gene signatures were genes that are known to be expressed in Tuft cells in the intestine and in the recently described Tuft-mTECs (**Figures 4B, C**, **5C**) (15, 20, 27). We observed many of the Tuft-cell signature genes to be bound by Fezf2 +/- 5000 bp of the TSSs, such as Il25, Trpm5, Reep5, Alox5, Tas1r3, Tas1r1, Plcb2, Dclk1, Tuba1a, and more. In the case of Tas2r138, a taste receptor encoding gene, the signal distribution analysis showed peaks for Elf3, Fezf2, and Klf4 around the TSS (**Figure 5C**). Additionally, Ehf was identified as one of the transcription factors regulated by Fezf2 (**Figures 4B, C**, **Supplementary Figure 5B**). Interestingly, the transcription factor Pou2f3, which is involved in Tuft-mTEC development also showed a Fezf2 ChIP-seq peak in proximity to the Pou2f3 gene; however, the peak was located at the 3´end of the Pou2f3 coding region and not in a promoter region upstream of the TSS (**Figure 5C**). As other genes are located in this region as well, the ChIP peak might reflect Fezf2 binding to those gene promoters instead.

Furthermore, we observed apoptosis-related genes to be enriched, among those, Gadd45α, β, and γ (Growth Arrest and DNA Damage genes), playing a role in DNA-damage response and DNA demethylation. Furthermore, components of the apoptosis signaling pathway, such as Fas, Myc, Bad, Bik, Bax, and Bcl-2 and the Caspases 3,7, and 9 were targeted by Fezf2 (**Figure 4E**, **Supplementary Figure 5C**). Fezf2 ChIP peaks did not show a preferential binding of Fezf2 at TRA genes +/- 5000 bp of the TSSs (**Supplementary Figures 5D, E**).

The transcription factor binding site databases TRANSFAC and JASPAR did not list positional weight matrices for the transcription factor Fezf2; thus, we aimed to use our Fezf2 ChIP-seq data to extract the putative binding sequences from the peaks to identify the binding motif of Fezf2. We extracted sequences from 50 bp up- and downstream of the peak summits from MACS2 and used MEME suite to identify enriched motifs. A previous study on Fezf2-dependent gene regulation used a zebrafish model to predict the Fezf2 binding motif (42). Using STAMP, we compared the predicted motifs from the zebrafish study to our mouse Fezf2 ChIP-seq-derived sequence motifs. Thereby, we identified one of the predicted Fezf2 binding motifs found in MEME (E-value 5.5e-008, 3456 associated sites) to be statistically similar to the MEME-motif from Chen et al. using STAMP analysis (E-value 2.0902e-02) (**Figure 5D**).

### 2.5 Fezf2 regulates mTEC maturation and Tuft-mTEC development

**Figure 6:**
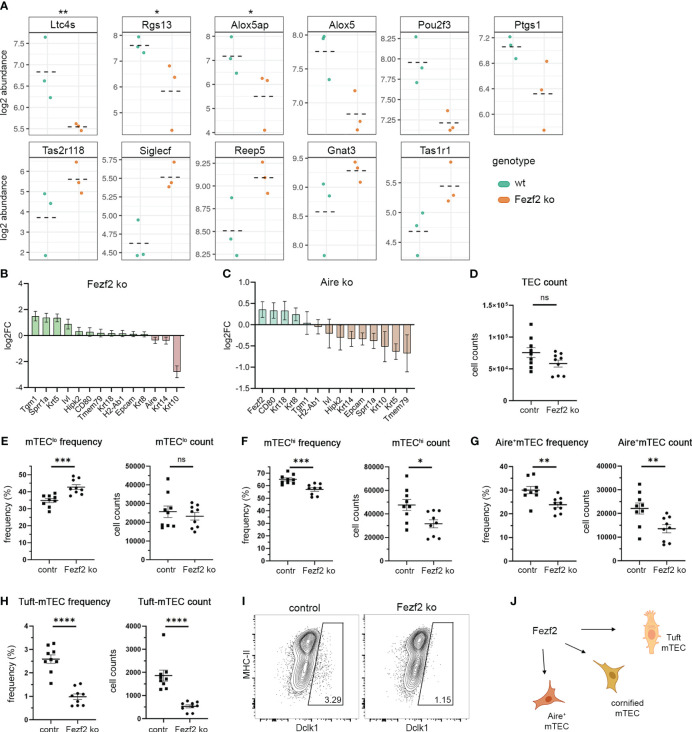
Fezf2 regulates maturation and Tuft cell gene signatures in mTECs. **(A)** Gene expression plots indicate the log2 abundance of transcript for Tuft cell signature genes, which are differentially expressed in the Fezf2 ko compared to the wt RNA-seq dataset by Tomofuji et al.; wt (green), Fezf2 ko (orange), dashed lines indicate the mean; upper panel: down-regulated genes; lower panel: up-regulated genes; **≤0.05; *≤0.1 **(B)** Gene expression plots indicate the log2FC of transcript for keratinization and maturation marker genes in Fezf2 ko mice compared to wild-type mice. Mean +/- SD. **(C)** Gene expression plots indicate the log2FC of transcript for keratinization and maturation marker genes in Aire ko mice compared to wild-type mice. Mean +/- SD. **(D)** FACS analysis of FoxN1-cre/Fezf2-flox mice, comparing FoxN1-cre^neg^/Fezf2-floxed (cre^-^) to FoxN1-cre^pos^/Fezf2-floxed (cre^+^) mice for their absolute TEC numbers (CD11c^neg^CD45^neg^EpCAM^pos^), **(E)** mTEC^lo^ (CD45^neg^EpCAM^pos^Ly51^neg^MHCII^low^) frequency and absolute numbers, **(F)** mTEC^hi^ (CD45^neg^EpCAM^pos^Ly51^neg^MHCII^high^) frequency and absolute numbers, **(G)** Aire^pos^ mTEC (CD45^neg^EpCAM^pos^Ly51^neg^Aire^pos^) frequency and absolute numbers, **(H)** Dclk1^pos^ Tuft-mTEC (CD45^neg^EpCAM^pos^Ly51^neg^Dclk1^pos^) frequency and absolute numbers, depicted are the results from two independent biological replicates with 4-5 mice per experiment. Statistics are calculated using unpaired Student’s t-test +/- SEM. **(I)** Representative FACS plots of Dclk1^pos^ Tuft-mTEC analysis for control (left) and Fezf2 ko (right) mice. **(J)** Model of the regulatory role of Fezf2 on Tuft-mTEC gene signature expression and cell development, on cornified mTEC gene signature expression, and on Aire^+^mTEC gene signature expression and cell development. See also **Supplementary Figure 6** and **7**. ns (P > 0.05), * (P ≤ 0.05), ** (P ≤ 0.01), *** (P ≤ 0.001), **** (P ≤ 0.0001).

Taking together these results, the Fezf2 and Ehf ChIP-seq targets showed features known from late mTEC development, post-Aire mTEC, and Tuft-mTEC stages. Notably, both Fezf2 and Ehf showed an implication in the regulation of late mTEC developmental signatures; however, the Tuft-mTEC signatures were specific to the Fezf2 target genes and not represented in the Ehf ChIP-seq peaks. The role of Fezf2 in the regulation of gene expression in the recently identified Tuft-mTECs has not been previously described. Therefore, we analyzed available RNA sequencing data from Fezf2-ko mice (GSE144877) (22) to analyze the gene expression levels of Tuft cell signature genes in the Fezf2 knockout (**Figure 6A**). Several of the Tuft-cell signature genes were differentially expressed in the Fezf2 ko compared to wild-type RNA-seq data sets. Notably, as described by the group of Takayanagi, Fezf2 can regulate gene expression in mTEC positively or negatively, depending on the gene and other co-regulators. We observed Ltc4s, Rgs13, Alox5ap, Alox5, Pou2f3, and Ptgs1 to be downregulated in the Fezf2 knockout, whereas Tas2r118, Siglecf, Reep5, Gnat3, and Tas1r1 rather showed a tendency to be upregulated in the Fezf2 knockout. We were wondering how late-mTEC development post-Aire and Tuft-mTEC gene signatures might arise and how they might be intertwined. Hence, we also analyzed the Aire knockout RNA-seq data of the GSE144877 data set and compared the effects of the Aire and Fezf2 knockout on maturation and Tuft-mTEC gene signature expression (**Figures 6B, C**, **Supplementary Figures 6A, B**). Further, we explored diversity indices as a measure of the effect of the Fezf2 and Aire knockout on gene diversity. We analyzed the diversity of all genes, non-TRAs, and TRAs in both knockout and control samples of Aire and Fezf2 using the Shannon-Weaver index and the inverse Simpson index (**Supplementary Figures 6C, D**). When comparing the Aire knockout to the control, both indices indicated a reduction in diversity across all gene categories. However, in the case of Fezf2 knockout compared to its control, only the inverse Simpson index demonstrated reduced diversity, and this was specifically observed in the non-TRA genes.

The regulatory influence of Aire on Tuft gene expression affected fewer genes compared to the impact of Fezf2. However, we did observe that some Tuft cell signature genes were positively regulated by Aire and thus downregulated in the knockout, including Gnat3, Avil, and Tas2r118. Conversely, other genes such as Siglecf, Tuba1a, Dclk1, and Il17rb appeared to be negatively regulated by Aire. Noteworthy, Siglecf was downregulated in the Aire ko (positively regulated by Aire) but upregulated in the Fezf2 ko (negatively regulated by Fezf2). However, Gnat3 and Tas2r118 were both upregulated in the Aire ko and the Fezf2 ko; thus, both genes were negatively regulated by Aire and Fezf2. The Fezf2 ChIP-seq data showed direct binding of Fezf2 to Tuft-cell signature genes and, in conjunction with the Fezf2 knockout analysis, identified a direct regulation of Tuft cell gene signature in mTECs by Fezf2. The Aire-dependent regulation of Tuft-cell genes, however, might be an indirect regulation through other TFs, which, themselves, are regulated by Aire.

Next, we analyzed the effect of the Fezf2 ko on the gene expression levels for mTEC developmental marker genes, keratinization, and cornification genes, which showed Fezf2 peaks in the ChIP-seq analysis (**Figures 5A, B**, **6B**). Tgm1, Sprr1a, Krt5, and Ivl were upregulated in the Fezf2 ko; thus, their expression is repressed by Fezf2, whereas Krt10 was downregulated in the Fezf2 ko. Hence, the gene expression of Krt10 is positively regulated by Fezf2 in mTECs. We also analyzed the effect of the Aire ko on the gene expression levels of those genes and found Aire to repress Fezf2 expression and Sprr1a, Krt5, and Krt10 to be positively regulated by Aire, leading to a downregulation in the ko (**Figure 6C**). Notably, the observed changes in gene expression for maturation and Tuft-cell signature genes in the Fezf2 and Aire-ko sequencing datasets were moderate for some of the described genes. The sequencing in those datasets was performed on total mTECs, not separating immature, mature, and late mTECs. The Tuft cell gene expression, however, is restricted to mature and late-mTECs, i.e., Tuft-mTEC subsets. Due to the heterogeneity of the analyzed total mTEC population, the effect of the Fezf2 knockout on Tuft-cell gene expression and late mTEC developmental stages is probably underrepresented in this analysis. Therefore, we used conditional knockout mice for Fezf2 by crossing Fezf2-flox mice to the epithelial-specific cre mouse line Foxn1^ex9cre^ to analyze the effect of the Fezf2-deficiency on mTEC cellularity. The overall TEC numbers in Fezf2-ko mice (FoxN1-cre^pos^/Fezf2^floxflox^) compared to control mice (FoxN1-cre^neg^/Fezf2^floxflox^) were not significantly affected by the Fezf2-deficiency (**Figure 6D**). However, analyzing the two subpopulations of mTEC^lo^ (CD45^neg^EpCAM^pos^Ly51^neg^MHCII^low^) and mTEC^hi^ (CD45^neg^EpCAM^pos^Ly51^neg^MHCII^high^) separately revealed a reduction of mTEC^hi^ in frequency and absolute numbers, while the mTEC^lo^ absolute cell count was unaffected, resulting in an increase in the relative mTEC^lo^ frequency (**Figures 6E, F**). Further, Aire^pos^-mTECs were affected by the Fezf2 knockout, leading to a 39% reduction (fold change of 1.6 contr/ko) in absolute numbers and a reduction in frequency of Aire^pos^-mTECs from 30% of mTECs (22110 ± 2351 number of cells) to 23.8% of mTECs (13504 ± 1696 number of cells) (**Figure 6G**). Moreover, we observed a severe reduction of thymic Tuft-cells in the Fezf2 knockout, leading to a reduction of 71% (fold change of 3.5 contr/ko) in absolute numbers and a reduction in frequency of Dclk1^pos^-mTECs from 2.6% of mTECs (1861 ± 236 number of cells) to 1% of mTECs (527 ± 67 number of cells) (**Figures 6H, I**). Thus, the analysis of the Fezf2 knockout in TECs revealed a role of Fezf2 in the development of Aire^pos^-mTECs and Dclk1^pos^-Tuft-mTECs.

In summary, we set out to reveal the transcriptional network responsible for the gene regulation in mTECs and the resulting mosaic expression patterns in TRA-specific mTEC subsets using a combination of comparative RNA-seq, ATAC-seq, and footprinting prediction analysis. We used ChIP-seq analysis and gene knockout studies to validate the regulatory role of gene expression in mTECs and identified transcription factors of the ELF, ESE, ERF, and PEA3 subfamily of the ETS transcription factor family and members of the Krüppel-like family of transcription factors to be involved. The transcription factors Ehf, Elf3, Klf4, and Fezf2 bind to the TSS of genes characteristic for late mTEC development and the Tuft-mTEC stage in particular. Comparing the expression levels of those transcription factors across tissues, based on the mouse ENDCODE transcript data, and across different immune cell types, based on mouse Immgen transcript data, Fezf2 shows the highest specificity and restricted expression to brain tissues and mTECs in the thymus, followed by Elf3, which is only reported in mTECs in the Immgen database but expressed in multiple tissues, based on the ENCODE database (**Supplementary Figure 7**). In comparison, Ehf is expressed in multiple cell types, such as B cells, DCs, splenic basophil (Ba_Sp), and stromal cells in subcutaneous lymph nodes (IAP_SLN), thymic epithelial cells, and in multiple tissues such as intestine, bladder, stomach, colon, duodenum, ovary, genital fat pad, kidney, and lung, but not detected in the thymus, which might be due to the sensitivity of the RNA-seq and the restricted expression in thymic epithelial cells. Of those four transcription factors analyzed, Klf4 showed the broadest expression pattern in multiple tissues and cell types.

## 3 Discussion

Regarding the transcriptional regulation of the self-peptide gene expression patterns in mTECs, it is known that: I. The transcription factors regulating gene expression in peripheral tissues are dispensable for the corresponding gene regulation in the thymus. II. Gene co-expression patterns comprise genes of different tissue origins and molecular functions. III. Epigenetic and miRNA-based regulation of gene expression plays a role in the regulation of promiscuous gene expression. IV. Aire and Fezf2 are regulating part of the TRA repertoire expression in mTECs. We set out to analyze late mTEC development to shed light on the TRA expression in mature and late mTEC stages using ATAC footprinting analysis as an unbiased genome-wide screen for putative regulators and ChIPmentation sequencing to analyze their functional relevance (27). Although SC techniques steadily improve with regard to sequencing depth and uniform quality performance, some approaches still encounter limitations. While ATAC sequencing became feasible on the single cell level (43, 44), drop-outs in SC-seq in combination with the chromosomal distribution of reads are limiting factors for reliable and robust ATAC footprinting analysis.

Therefore, we used ATAC sequencing and footprinting analysis on Tspan8-expressing mTEC subsets instead (11, 12). Based on the previously postulated lineage bifurcation and sliding co-expression models that describe possible scenarios of the developmental origins of the TRA mosaic expression patterns, we wanted to use a TRA-positive mTEC subset to apply our analysis to. This allows us to reduce the heterogeneity of the mTEC population and to analyze regulatory mechanisms within TRA-positive subsets instead. We chose Tspan8 as an exemplary TRA for this purpose as this TRA is expressed in MHCII^low^ and MHCII^high^ mTECs, allowing for additional developmental sub-setting of the TRA-positive subpopulation.

This approach enabled us to investigate the transcription factors regulating promiscuous gene expression in murine medullary thymic epithelial cells in an unbiased and genome-wide manner, leading to the identification of multiple transcription factors of the ELF, ESE, and PEA3 subfamily of the ETS transcription factor family, and members of the Krüppel-like family of transcription factors to be involved in late mTEC development and the regulation of promiscuous gene expression. Using ChIPmentation and RNA-seq profiling, we validated the binding of the identified transcription factors to the promoter regions of mTEC gene signatures. We extended our ChIPmentation analysis to include Fezf2, a transcription factor regulating gene expression signatures in mTECs. Surprisingly, we found Fezf2 and Ehf to regulate late mTEC developmental gene signatures in mTECs and gene signatures specific to the recently identified thymic Tuft cells to be regulated by Fezf2. Thymic tuft cells were shown to share several features with tuft cells from the mucosal barriers in the airways and the intestine, such as the expression of canonical taste receptor transduction pathways and Il25 secretion (15, 20, 29, 30). However, Tuft-mTECs were shown to be special in regards to their spatial localization next to cornified structures in the thymus and a developmental lineage that at least partially passes through an Aire-expressing mTEC stage, which is described to be dependent on the Aire-interaction partner Hipk2 (20, 45). Fezf2 was originally described as a transcription factor with neurodevelopmental regulatory function in zebrafish and Xenopus (46–48). The group of Takayanagi identified Fezf2 as a transcription factor involved in the regulation of some Aire-dependent but mostly Aire-independent TRA gene expression in murine mTECs (21, 22). In their original study, they used microarrays and focused their microarray analysis on CD45-EpCAM+CD80hi mTECs, which nowadays, we know to characterize mature mTECs, excluding earlier developmental stages, which are characterized by low CD80 and MHC expression levels, and later post-Aire mTECs, which downregulate CD80 and MHC again (21). In a recent study by the same group, they used RNA sequencing on CD45-EpCAM+Ly51-UEA1+ mTECs and compared Fezf2- and Aire-dependent gene regulation in mTECs (22). They described Fezf2 to be expressed in 30% of the MHCII low and in approximately 90% of the MHCII high mTEC developmental stages.

The Fezf2-dependent development of Tuft-mTECs discovered in this study identifies an alternative role of Fezf2 during mTEC development in which Fezf2 regulates the development of late developmental mTEC stages and the Tuft-mTEC stage, thereby indirectly affecting the representation of TRA-groups, which are normally expressed and presented by those mTEC subsets. We did not observe a tendency for Fezf2 to primarily regulate TRA genes in mTECs (**Supplementary Figures 5D, E**) but rather an enrichment for developmental gene signatures and late mTEC markers to be enriched among the Fezf2 targets in the ChIPmentation-seq analysis. This conclusion coincides with the observation that Fezf2-dependent genes are not restricted to small subsets of mTECs and are not restricted to co-expression groups as observed in the mosaic expression patterns of thymic TRAs (22). Interestingly, the Aire and Fezf2 knockouts exhibited variations in diversity indices, which may indicate distinct mechanisms of gene expression regulation (**Supplementary Figures 6C, D**). Understanding the specific involvement of Fezf2 in TRA gene regulation continues to be an area requiring further exploration, indicating a clear need for additional investigative efforts to delineate its role accurately (49). Further, we found Fezf2 to bind to the Hipk2 promoter in our ChIPmentation sequencing (**Figure 5A**). In a previous study, we showed that Hipk2 is involved in Tuft-mTEC development as the FoxN1^cre^/Hipk2^fl/fl^ conditional knockout of Hipk2 led to a reduction of Tuft-mTEC numbers (20). In this study, we identified a regulatory cascade in which Fezf2-dependent expression of Hipk2 may be one of the ways through which Tuft-mTEC development is regulated. Interestingly, the reduction of Tuft-mTEC numbers in the conditional Hipk2-ko was around 60%, congruent with the reduction of Tuft-mTEC numbers in the conditional Fezf2-ko (**Figure 6H**). Hence, Fezf2 is the third identified transcription factor involved in thymic tuft cell development to date, besides the transcription factors Pou2f3 and Sox4, which were described to influence tuft cell development in the thymus (20, 50). This novel role of Fezf2 in the regulation of late mTEC and Tuft-mTEC subset development further dismantles the mysteries of promiscuous gene expression complexity in the context of central tolerance induction.

Based on findings from previous studies, which described Fezf2 to be involved in the development of corticospinal neurons and sub-cerebral projection neurons in the brain (51), we speculate that Fezf2 might be involved in the formation of Tuft-cell neurofilaments in the thymus putatively regulated in an LTßR and Traf6-dependent manner as Fezf2 was shown to be regulated through LTßR and Traf6 signaling (21). The typical Tuft-cell morphology is characterized by apical microvilli containing F-actin microfilaments and Tuft-cells are described to further contain neurofilaments, which in the brain characterize mature neurons (52).

J. Abramson and colleagues previously described the histone deacetylase 3 (Hdac3) to be involved in mTEC-specific developmental program regulation (23). Interestingly, they identified Pou2f3, Ascl1, Fezf2, and Ehf among the top genes induced by HDAC3. Reassessing those findings, these data imply a role of Hdac3 not only in mTEC development but likely in Tuft-mTEC development as well. Moreover, they assumed Hdac3 to act through Notch repression and described a small Notch-positive TEC subpopulation of approximately 6% within the mTEC low compartment. Furthermore, Notch1 overexpression led to repression of Ascl1, Fezf2, and Pou2f3. Speculatively, hinting toward a regulatory cascade in which Tuft-mTECs might be dependent on Fezf2 and Pou2f3, which are regulated by Hdac3-mediated repression of Notch1. Detection of Ehf expression in mature mTECs was previously reported using single-cell RNA sequencing analysis (referred to as TEC3 (53)). Furthermore, Ehf expression could also be detected in human thymus sample mTECs using bulk-RNA-sequencing of sorted mTECs (54), supporting the notion that Ehf serves as a fundamental transcriptional regulator in mTEC maturation across species.

In contrast to the current understanding of promiscuous gene expression in the thymus, which is characterized by its mosaic expression patterns of self-peptides in mTECs, irrespective of the tissue origin of the peptides, not resembling the peripheral organ-specific co-expression patterns; thymic Tuft cells are different to this end. Tuft-mTECs show a strong phenotypical resemblance with peripheral tuft-cells, with a polarized, ciliated structure. Moreover, they are capable of contributing to self-tolerance induction; however, they seemingly undertake this in difference to the phenomenon of promiscuous gene expression by peripheral tissue mirroring and resemblance of the peripheral cell type. Alternatively, there might be an additional role of the tuft cell morphology and sensing capabilities of Tuft-mTECs with regards to infections, as described for the mucosal barrier tuft cells (55–58). Yet, infections within the thymic tissue have not been described so far, hence the chemosensory function of thymic tuft cells requires further investigation.

In human primary keratinocytes, Klf4 was shown to drive epidermal differentiation through the acquisition of H3K27ac and the establishment of enhancer-promoter contacts at enhancers of differentiation-associated genes. Moreover, the same study described Ehf as an essential regulator of keratinocyte differentiation using genome-wide enhancer profiling for TFBM and knockdown studies in organotypic human epidermal tissue (59). The epithelium-specific ETS (ESE) transcription factors ELF3, ELF5, and EHF have been shown to be important regulators of epithelial tissue homeostasis and cancer in multiple tissues such as the gastrointestinal tract (esophagus, large and small intestine, and stomach), salivary gland, bladder, liver and prostate for ELF3, salivary gland, breast and bladder for ELF5 and salivary gland, esophagus, vagina, prostate, colon, skin, bladder, cornea, and breast for EHF (60–63). Here, we show that Ehf and Elf3 TFBMs are enriched in genes expressed in late developmental mTEC stages, characterized by Tspan8 expression and upregulation of the keratinization markers involucrin and keratin 10 (**Figure 3**, **Supplementary Figure 1**). We observe the Ehf transcription factor to bind to promoter regions of mTEC developmental marker genes, such as Epcam, Aire, and CD80 as well as late developmental, keratinization, and cornification-associated genes such as Krt5, Krt14, Sprr1a, and Klf4 (**Figures 4**, **5**). Interestingly, Klf4 was previously shown to be targeted by EHF using ChIP-seq on cornea epithelium (63). Thus, the identified regulatory network composed of the ELF, ESE, and PEA3 subfamily of the ETS transcription factor family, and members of the Krüppel-like family of transcription factors might be a general feature of epithelial cell development in different tissues, including thymic epithelial cells.

In recent years, a growing number of mTEC subsets were identified and characterized, revealing that the TEC compartment is highly diverse and functionally compartmentalized. Beyond the veil of this high diversity of mTEC subpopulations (early Ccl21^pos^ mTECs, mature MHCII^high^, Aire^pos^, and Aire^neg^ mTECs, Krt10^pos^ late mTECs, Dclk1^pos^ Tuft-mTECs) and the additional high complexity of self-peptide co-expression patterns lays a comparably complex transcriptional network of regulatory factors involving chromatin remodeler, transcription factors, and epigenetic modifiers. In the case of Tas2r138, a taste receptor encoding gene, the signal distribution analysis showed peaks for Elf3, Fezf2, and Klf4 around the TSS, suggesting a combinatorial gene transcriptional regulation in Tuft-mTECs (**Figure 5C**). These results favor the model of a multifactor network regulating self-peptide expression in central tolerance rather than stochasticity and further extend the mechanistic knowledge of the regulation beyond the distinction of Aire-dependent and Aire-independent genes.

## 4 Materials and methods

### 4.1 Mice

The BL6N6 wild-type mice used in this study were purchased from Charles River Laboratories (stock no: 027) and were used at 4–6 weeks of age. B6(Cg)-Foxn1tm3(cre)Nrm/J (stock no: 018448) mice were obtained from The Jackson Laboratory and Fezf2-flox mice were kindly provided by Nenad Sestan (Yale University) (64). Breeding and cohort maintenance of FoxN1-cre/Fezf2-flox mice were performed in the animal facility of UCSF Diabetes Center. Mice were housed in a specific pathogen-free and viral antibody-free animal facility in accordance with the guidelines established by the Institutional Committee on Animal Use and Care (IACUC) and Laboratory Animal Resource Center (LARC). Control and experimental mice were co-housed. All mice were 4-6 weeks of age. Mice were housed in 12/12 hrs light/dark cycles (6 am to 6 pm light) at a 70~72 F degree temperature range, and relative humidity within 40~50%.

### 4.2 Medullary thymic epithelial cell preparation

Primary mTECs from mice were isolated by sequential fractionated enzyme digestion as previously described (12). Mouse thymi were cut into pieces and digested in two rounds of collagenase mix (RPMI, 10 mM Hepes, 2% FCS, 0.2 mg/ml Collagenase Typ IV) under magnetic stirring for 10 min at 37°C in a water bath under magnetic stirring, followed by four to five digestion rounds in a collagenase/dispase enzyme mix (RPMI, 10 mM Hepes, 2% FCS, 0.2 mg/ml Collagenase Typ IV, 0.2 mg/ml and 6 U/mg Dispase, 25 μg/ml DNAse I) for 20 min at 37°C in a water bath under magnetic stirring. The collagenase/dispase cell fractions were pooled and filtered through a 70 µm cell strainer. After digestion, the single cell fraction was pre-enriched for thymic stromal cells by depleting CD45 positive cells using anti-CD45 magnetic beads and the autoMACS (Miltenyi Biotec).

Pre-enriched stromal cell fractions were stained using the following mAbs: anti-CD45-Per-CP (clone 30-F11, BD Pharmingen), anti-EpCAM-A647 (G8.8 hybridoma (65);), anti-I-A(b)-FITC (clone AF6-120.1, BD Pharmingen) or anti- I-A/I-E-PE (clone M5/114.15.2, BD Pharmingen), anti-CDR1-PB (CDR1 hybridoma (66);), anti-Tspan8-PE (clone 657909, R&D Systems), isotype control rat-IgG2b-PE (eBioscience), DCLK1 (Abcam polyclonal ab31704), DCLK1 (EPR6085) Ly51-PE (6C3), CD11c- Pe-Cy7 (N418, Biolegend), AIRE- A488 (5H12, Biolegend). Dead cells were excluded using propidium iodide in a final concentration of 0.2 µg/ml for cell sorting or Live/Dead Fixable Blue Dead Cell Stain (Thermo Fisher) for FACS analysis on an LSRII. Cells were sorted on an Aria II cell sorter (BD) or data was collected on a LSRII Flow Cytometer (BD Biosciences). FACS data was analyzed using FlowJo 10.3 software (TreeStar Software).

### 4.3 Total RNA preparation

Total RNA from sorted mTECs was isolated and purified using the High Pure RNA Isolation Kit (Roche Diagnostics) according to the manufacturer’s protocol and used for RT and qPCR or RNA sequencing.

### 4.4 Quantitative real-time PCR

For quantitative real-time PCR (qPCR), total RNA was reverse transcribed into cDNA using random primers and Superscript II Reverse Transcriptase (Invitrogen) following the manufacturer’s protocol.

Real-Time PCR was performed in a total volume of 20 µl using Power Sybr Green Mix (Applied Biosystems) and the ViiA 7 real-time PCR machine (Applied Biosystems). Intron-spanning primers were designed using Primer3 software (67). Reactions were performed in technical duplicates and biological replicates, as indicated in the respective plots. Values were normalized to Actin expression and to total thymus cDNA using the δδCT method.

### 4.5 RNA sequencing

Total RNA was extracted from FACS-isolated mTECs, being Tspan8^pos^ or Tspan8^neg^ and MHCII^low^ or MHCII^high^. RNA sequencing libraries were prepared as follows: 1 µl of a 1:1,000,000 dilution of ERCC Spike-In Mix (Life Technologies) in RNase-free water was included in a total volume of 5 µl lysis buffer. During analysis, sequencing reads mapping to ERCC spike-ins were used for the estimation of technical noise levels and for calling of significantly highly variable genes by a published method (68). We used 19 cycles of initial PCR amplification and used a ratio of 0.6:1.0 (beads/total PCR volume) of Ampure XP beads (Beckman Coulter) for the first PCR purification to minimize primer dimer carryover. After the first PCR amplification, cDNA libraries were screened via quantitative PCR (we used a 1:10 dilution of purified cDNA libraries for quantitative PCR) for expression of a mouse housekeeping gene (*Ubc*), and the distribution of library size was checked on a Bioanalyzer instrument (Agilent). Only cDNA libraries that passed both quality controls were processed further. We used 100 pg of cDNA for the ‘tagmentation’ (transposase-based fragmentation) reaction and applied 12 cycles for the final enrichment PCR. The final purification step was performed with a ratio of 0.8:1.0 (as above) of Ampure SPRIselect beads (Beckman Coulter). The final ‘multiplexed’ sequencing libraries were sequenced on a HiSeq 2500 machine (Illumina) using 54 bp single-end sequencing. Raw reads were assessed for quality using FastQC. Reads were aligned to the mouse reference transcriptome (Gencode vM10) using Salmon (v1.8.0) (69). Data normalization and differential gene expression analysis were performed using DESeq2 (v1.30.0) (70) in R.

### 4.6 ATAC sequencing

ATAC-seq experiments were performed as reported (71) with the following modifications: murine-sorted mTECs were used for ATAC-seq experiments, being Tspan8^pos^ or Tspan8^neg^ and MHCII^low^ or MHCII^high^. We used 50% of each purified ‘tagmentation’ reaction for enrichment PCR (without five cycles of pre-amplification). Each enrichment PCR was monitored individually with the StepOnePlus Real-Time PCR System (Life Technologies) and the amplification reaction was stopped as soon as amplification approached saturation. After the enrichment PCR and subsequent purification of PCR products, gel extraction was performed (QIA MinElute Gel Extraction Kit; Qiagen) to remove primer dimers. The final ‘multiplexed’ sequencing libraries were quantified by quantitative PCR and were sequenced on a HiSeq 2500 machine (Illumina) using 105 bp paired-end sequencing. Raw reads were assessed for quality using FastQC. ATAC reads were aligned to the mm10 genome using Bowtie2 (72) with the following settings: –very-sensitive -k 4 -X 1000. Mitochondrial reads, multimapping reads, low-quality reads, and non-unique alignments were removed using SAMtools with the following settings: -F 1804 -f 3 -q 30. PCR duplicates were removed using Picard’s MarkDuplicates. Peak-calling was performed using MACS2 with the following settings: -g mm –broad –broad-cutoff 0.01 -f BAMPE. Subsequently, consensus peaks were generated by merging called MACS2 peaks using bedtools merge. The resulting peak regions were annotated using HOMER to identify the nearest genes and genomic features. Counts of reads mapped to consensus peaks were generated using featureCounts (73) with default settings. Differential ATAC peak analysis was performed using DESeq2 (70) in R. ATAC-seq signal distribution heatmaps were generated using deepTools (74) with the following settings: bamCoverage -bs 10 –normalizeUsing RPKM –ignoreDuplicates –extendReads. computeMatrix reference-point –referencePoint center –missingDataAsZero –binSize 10 –beforeRegionStartLength 1500 –afterRegionStartLength 1500. plotHeatmap –colorMap YlGnBu.

### 4.7 ChIP sequencing with tagmentation

Sorted mTECs were fixed in 1% formaldehyde for 8 min at room temperature. The fixation was stopped by adding glycine at a final concentration of 0.125 M. Nuclei preparation from cell pellets was performed using the Nexon method (nuclei extraction by sonication) (41) on a Covaris M220, using the following settings: Peak power 40%, duty factor 2.5, Cycles/Burst: 200, waterbath 7°C (min 5°C – max 9°C). The chromatin shearing was performed on a Covaris M220 using the following settings: Peak Power: 75, Duty Factor: 5.0, Cycles/Burst: 200, waterbath 7°C (min 5°C – max 9°C). DNA quantification and quality assessment were performed using Qubit (Thermo Fischer), BioAnalyzer (Agilent), or Tapestation (Agilent). Chromatin immunoprecipitation and tagmentation were performed using the ChIPmentation method according to the protocol described by Schmidl et al. (34). The following antibodies were used for chromatin Ips: anti-Klf4 (R&D, AF3158), anti-ZNF312 (Abcam, ab69436), anti-Ehf (Abcam, ab126963), and anti-Elf5 (Thermo Fischer, 720380). Library preparation was performed using Kapa HiFi HotStart Ready Mix (Roche) and Nextera custom primers (43). Libraries were sequenced on an Illumina NextSeq500. Data quality was evaluated using FASTQC (http://www.bioinformatics.babraham.ac.uk/projects/fastqc/), filtered and trimmed with Atropos (75), and mapped to the mm10 mouse genome build using BWA (76). After removing multi-mapping reads and duplicates, peaks were called MACS2 (77) with default parameters. Peak quality was evaluated with ChIPQC (78). Coverage and the reproducibility of peaks across replicates were evaluated with DeepTools. ChIPseeker (79) was used to annotate peaks to the closest transcriptional start site and perform functional enrichment analysis. Data was visualized using IGV (80). ChIP-seq signal distribution heatmaps were generated using deepTools (74) with computeMatrix reference-point –referencePoint TSS -b 5000 -a 5000 -R <bed files> -S <bigWig files> –skipZeros -o <output_matrix file> -p 4. plotHeatmap -m <output_matrix file> -o <output figure name> –colorMap Greens –outFileSortedRegions <output sortedRegions file> –regionsLabel <custom labels> –legendLocation upper-left. Signal density plots were generated using Spark (81) with the command python SparK.py -pr <coordinates> -cf <input bdg files> -tf <IP bdg files> -gff <reference transcripts file> -tg 1 2 3 4 -cg 1 2 3 4 -gl Ehf Elf3 Fezf2 Klf4 -l input IP -ps averages -scale no.

### 4.8 Fezf2-ko and Aire-ko RNA sequencing analysis

Raw data (fastq files) for the WT and Fezf2KO mTEC samples, from the study by Tomofuji et al. were downloaded from GEO (GSE144877). Raw reads were examined for quality issues using FastQC (http://www.bioinformatics.babraham.ac.uk/projects/fastqc/) to ensure library generation and sequencing were suitable for further analysis. Reads were processed to counts through the bcbio RNA-seq pipeline implemented in the bcbio-nextgen project (https://bcbio-nextgen.readthedocs.org/en/latest/). If necessary, adapter sequences, other contaminant sequences such as polyA tails, and low-quality sequences with PHRED quality scores less than five were trimmed from reads using cutadapt (82). Trimmed reads were aligned to UCSC build mm10 of the mus musculus genome (mouse), using STAR (83). Alignments were checked for evenness of coverage, rRNA content, genomic context of alignments (for example, alignments in known transcripts and introns), complexity, and other quality checks using a combination of FastQC and Qualimap (84). Counts of reads aligning to known genes were generated by featureCounts (73). In parallel, transcripts per million (TPM) measurements per isoform were generated by quasi-alignment using Salmon (69). Differential expression at the gene level was called with DESeq2 (70), preferring to use counts per gene estimated from the Salmon quasi-alignments by tximport (85). Quantitating at the isoform level has been shown to produce more accurate results at the gene level.

### 4.9 Fezf2 binding motif characterization

We extracted sequences from 50 bp up- and downstream of the peak summits from MACS2 and downloaded Fezf2 binding site sequences of zebrafish from Chen et al. (42). Using the same default parameters with max motif length set to 12bp, we used the MEME-suite (86) to detect the motifs in the sequences. We then calculated the statistical similarity between the motifs from the two studies using the STAMP (87). We further narrowed down the candidate motifs by MEME’s e-value significance and the number of sites contributing to the construction of the motif.

### 4.10 Transcription factor binding site identification

For all annotated genes (UCSC KnownGenes), the 2kb promoter upstream and 1kb downstream of the TSS were extracted and scored using the TRANSFAC collection of position weight matrices (PWM) and using the total binding affinity (TBA) method. Briefly, the TBA score is computed over the whole promoter sequence by summing for each position the maximum PWM score between the plus and minus strands (88). Then, for each PWM, the genes were ranked according to their TBA score in decreasing order. Using the ranked gene lists for a given set of genes, the recovery curve was determined for each PWM, and the area under the curve (AUC) was computed over the first 1,000 genes. AUC values were converted into a z-score by computing the mean AUC over all PWMs as well as the standard deviation. ATAC footprinting analysis was performed as previously described (33, 35).

## Data availability statement

The sequencing data reported in this paper have been deposited in the Gene Expression Omnibus (GEO) database, www.ncbi.nlm.nih.gov/geo (accession no: GSE232653 (RNA-seq), GSE234363 (ATAC-seq), and GSE232702 (ChIP-seq)).

## Ethics statement

The animal study was approved by Institutional Committee on Animal Use and Care (IACUC) and Laboratory Animal Resource Center (LARC) of the Harvard Medical School in Boston and University of California San Francisco. The study was conducted in accordance with the local legislation and institutional requirements.

## Author contributions

SL: Data curation, Formal Analysis, Investigation, Methodology, Software, Visualization, Writing – review & editing, analyzed the RNA-seq and ATAC-seq experiments. VB: Data curation, Formal Analysis, Investigation, Methodology, Software, Visualization, Writing – review & editing, analyzed ChIPmentation data and Fezf2 conventional knock-out RNA-seq data (GSE144877). PB: Data curation, Investigation, Methodology, Writing – review & editing, performed the RNA-seq and ATAC-seq experiments. CM: Data curation, Formal Analysis, Investigation, Methodology, Writing – review & editing, Visualization, performed and analyzed FoxN1-cre/Fezf2-flox conditional knock-out mouse FACS experiments. JY: Data curation, Formal Analysis, Investigation, Methodology, Software, Visualization, Writing – review & editing, performed the TFBM prediction for Fezf2. JB: Data curation, Formal Analysis, Investigation, Methodology, Writing – review & editing, performed and analyzed FoxN1-cre/Fezf2-flox conditional knock-out mouse FACS experiments. MA: Funding acquisition, Supervision, Resources, Investigation, Methodology, Writing – review & editing, FoxN1-cre/Fezf2-flox conditional knock-out mouse FACS experiments. SH: Funding acquisition, Supervision, Resources, Project administration, Data curation, Formal Analysis, Investigation, Methodology, Software, Visualization, Writing – review & editing, performed ChIPmentation data analysis, Fezf2 conventional knock-out RNA-seq data analysis (GSE144877) and performed the TFBM prediction for Fezf2. LS: Funding acquisition, Supervision, Resources, Data curation, Investigation, Methodology, Writing – review & editing, RNA-seq and ATAC-seq experiments UA: Funding acquisition, Supervision, Resources, Investigation, Writing – review & editing, ChIPmentation seq experiments KR: Funding acquisition, Supervision, Resources, Project administration, Conceptualization, Data curation, Formal Analysis, Investigation, Methodology, Visualization, Writing – original draft, Writing – review & editing, conceived the study, designed and performed experiments and analyzed the data of RNA seq, ATAC seq, ChIPmentation seq and FACS experiments.
