# Supplementary material for: Ehf and Fezf2 regulate late medullary thymic epithelial cell and thymic tuft cell development
Source: Front Immunol. 2024 Feb 14;14:1277365. doi: 10.3389/fimmu.2023.1277365 (PMC10901246; doi:10.3389/fimmu.2023.1277365)
Supplement: Supplementary file 1 [file DataSheet_1.pdf]

Supplementary Material

Ehf and Fezf2 regulate late medullary thymic epithelial cell and thymic tuft cell development

Authors:

Sören Lammers[1,†], Victor Barrera[2], Philip Brennecke[3,4], Corey Miller[6], Joon Yoon[2], Jared Balolong[6], Mark S. Anderson[6], Shannan Ho Sui[2], Lars M. Steinmetz[3-5], Ulrich H. von Andrian[7] and Kristin Rattay[7,8,*]

Affiliations:

[1] Institute for Theoretical Physics, Heidelberg University, Heidelberg, Germany.

[2] Bioinformatics Core, Harvard T.H. Chan School of Public Health, Boston, MA.

[3] Department of Genetics, Stanford University, School of Medicine, California, USA.

[4] Stanford Genome Technology Center, Stanford University, California, USA.

[5] European Molecular Biology Laboratory (EMBL), Genome Biology Unit, Heidelberg, Germany.

[6] University of California (UCSF) Diabetes Center, San Francisco, CA, USA.

[7] Division of Immunology, Harvard Medical School (HMS), 77 Avenue Louis Pasteur, Boston, USA.

[8] Pharmacological Institute, Biochemical Pharmacological Center, University of Marburg, Marburg, Germany.

[†] Current address: d-fine GmbH, 60313 Frankfurt, Germany

* Corresponding author: Prof. Dr. Kristin Rattay,

Pharmacological Institute, Biochemical Pharmacological Center, University of Marburg, 35032 Marburg, Germany

phone: (+49) 6421 28-25048; fax: (+49) 6421 28-65600, E-mail: kristin.rattay@uni-marburg.de

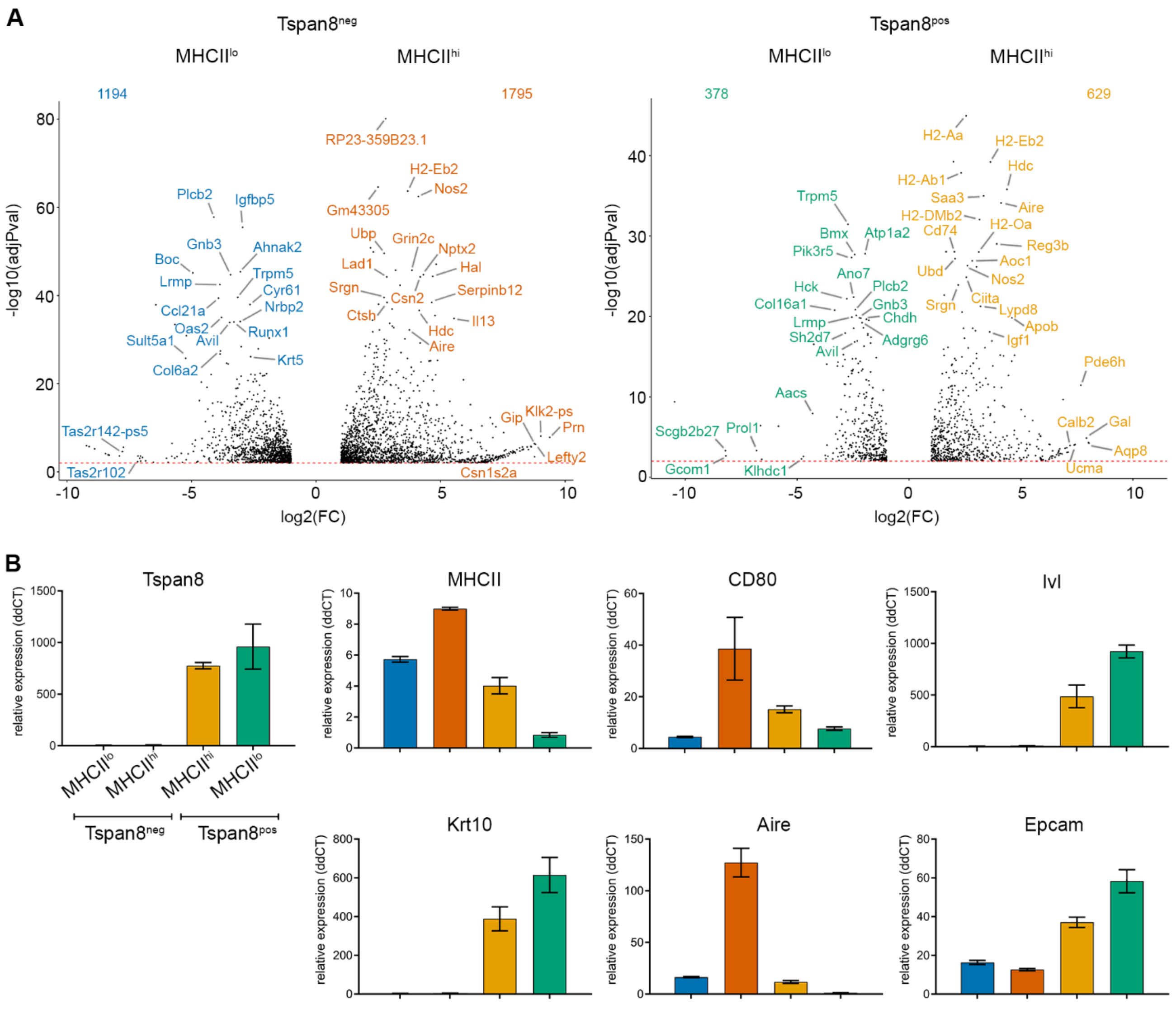

Supplementary Figure 1. Differential gene expressions from qRT-PCR validations and RNAseq analysis of MHCII^{lo} compared to MHCII^{hi} mTEC subpopulations. (**A**) Volcano plot of differential gene expression between Tspan8^{neg}MHCII^{lo} (blue) compared to Tspan8^{neg}MHCII^{hi} (red), left panel and Tspan8^{pos}MHCII^{lo} (green) compared to Tspan8^{pos}MHCII^{hi} (orange), right panel. (**B**) Expression values of Tspan8, MHCII (H2-Ab1), CD80, Ivl, Krt10, Aire and Epcam in Tspan8^{neg}MHCII^{lo} (blue), Tspan8^{neg}MHCII^{hi} (red), Tspan8^{pos}MHCII^{lo} (green) and Tspan8^{pos}MHCII^{hi} (orange) mTEC populations. Shown are the relative expression values obtained by quantitative real-time PCR, normalized to Actin and total thymus lysate (δδCT method), as mean ± SEM.

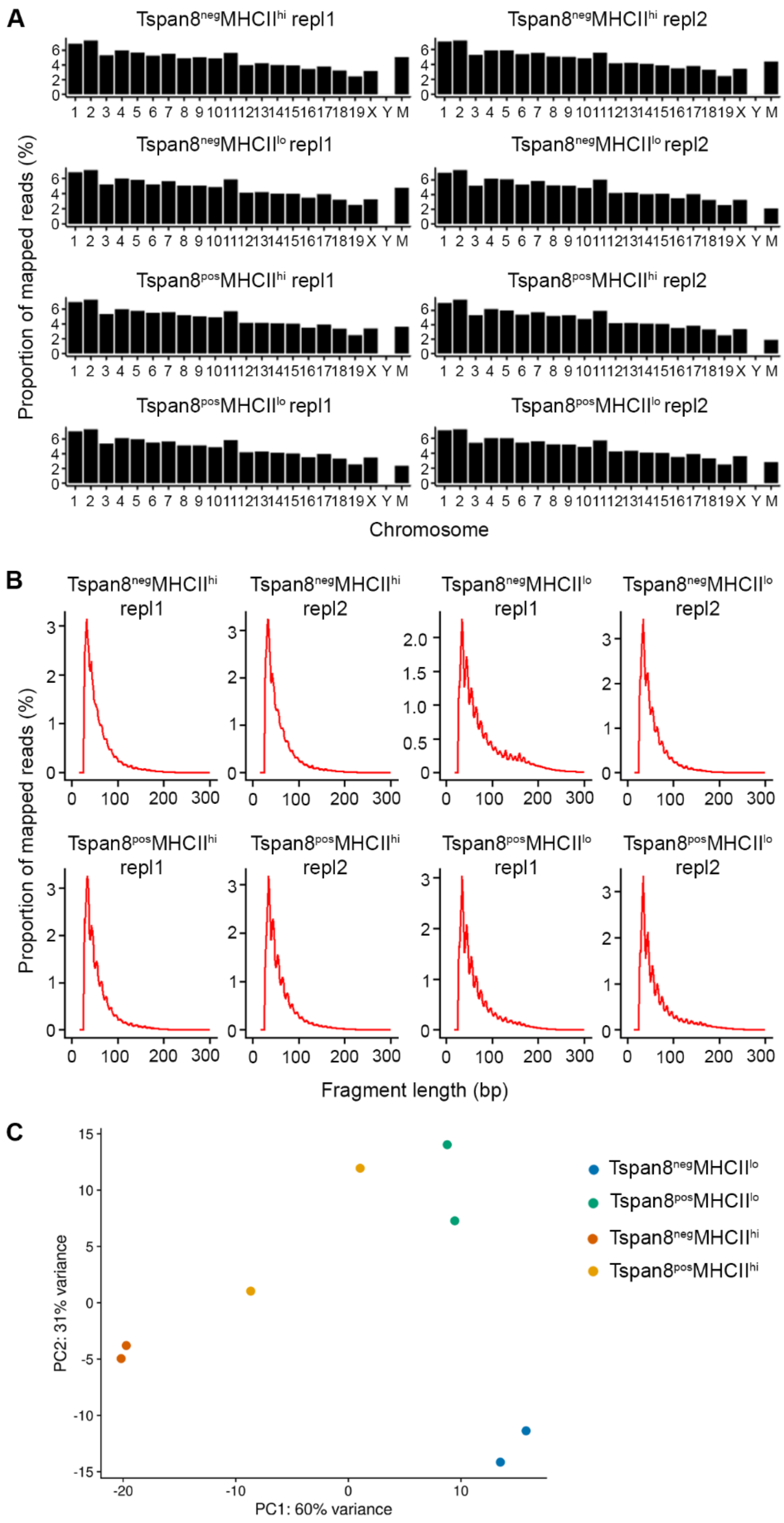

Supplementary Figure 2. Quality assessment of ATAC sequencing. (**A**) Chromosome distribution of aligned reads for ATAC sequencing of Tspan8^{neg}MHCII^{lo}, Tspan8^{neg}MHCII^{hi}, Tspan8^{pos}MHCII^{lo} and Tspan8^{pos}MHCII^{hi} mTEC populations. ATAC sequencing samples showed transposon insertions

in the mitochondrial chromosome (chrM) between 1.9-5.1 % of all mapped reads. (**B**) Fragment length distribution of ATAC sequencing reads. (**C**) Principal component analysis of Tspan8^{neg}MHCII^{lo} (blue), Tspan8^{neg}MHCII^{hi} (red), Tspan8^{pos}MHCII^{lo} (green), Tspan8^{pos}MHCII^{hi} (orange) mTEC populations from ATAC sequencing on top 1000 most variable consensus peaks.

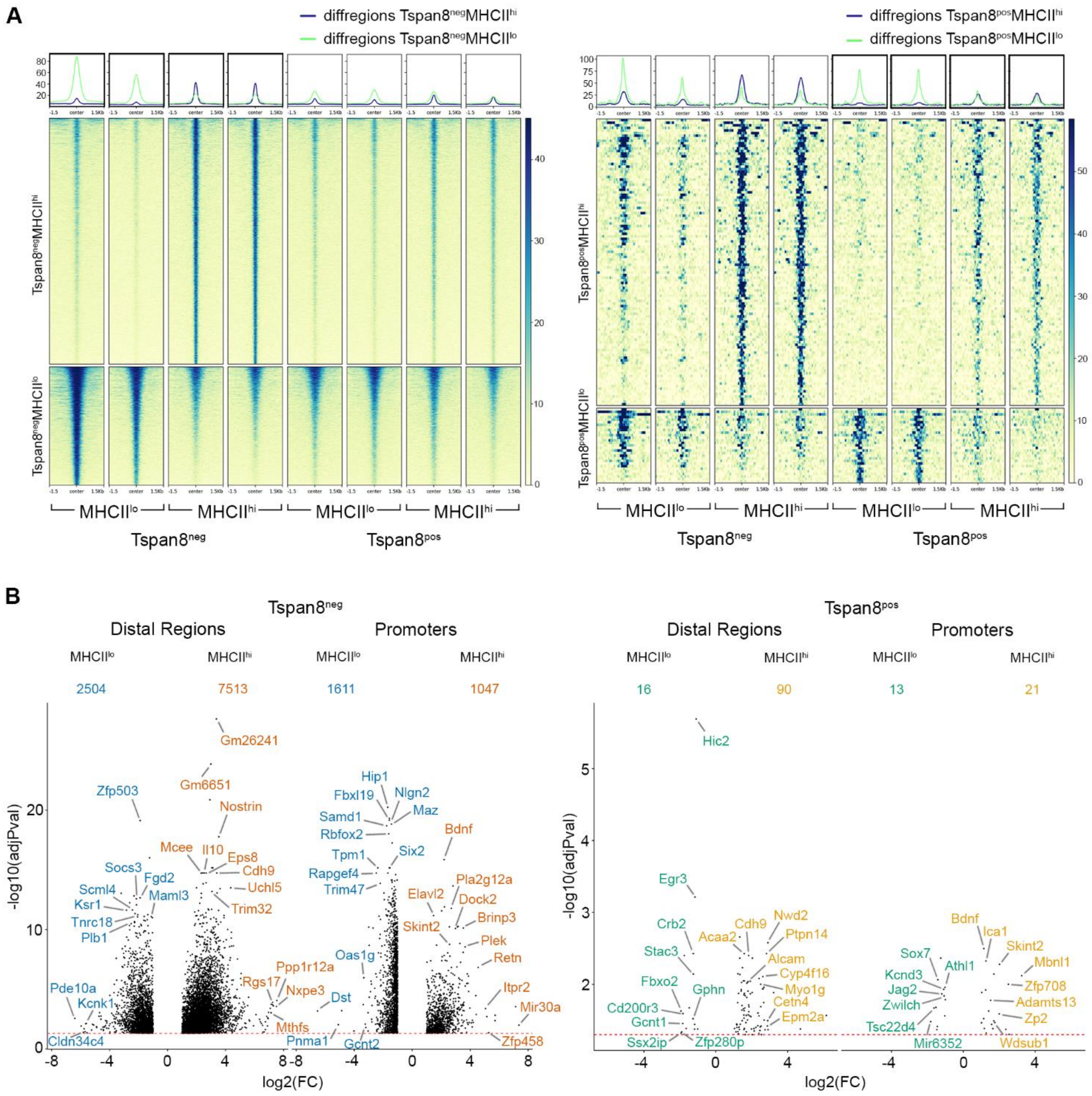

Supplementary Figure 3. Differential ATAC-seq footprints in MHCII^{lo} compared to MHCII^{hi} mTEC subpopulations. (**A**) Differential ATAC regions of Tspan8^{neg}MHCII^{lo} compared to Tspan8^{neg}MHCII^{hi} on the left and Tspan8^{pos}MHCII^{lo} compared to Tspan8^{pos}MHCII^{hi} on the right. Heatmaps show normalized signals within +/- 1.5 kb around the differential regions. (**B**) Volcano plot showing differential ATAC regions between Tspan8^{neg}MHCII^{hi} (red) compared to Tspan8^{neg}MHCII^{lo} (blue), left panel and Tspan8^{pos}MHCII^{lo} (green) compared to Tspan8^{pos}MHCII^{hi} (orange), right panel. The nearest genes to the differential distal regions and promoter regions are depicted. Numbers indicate the total amount of genes identified for each classification.

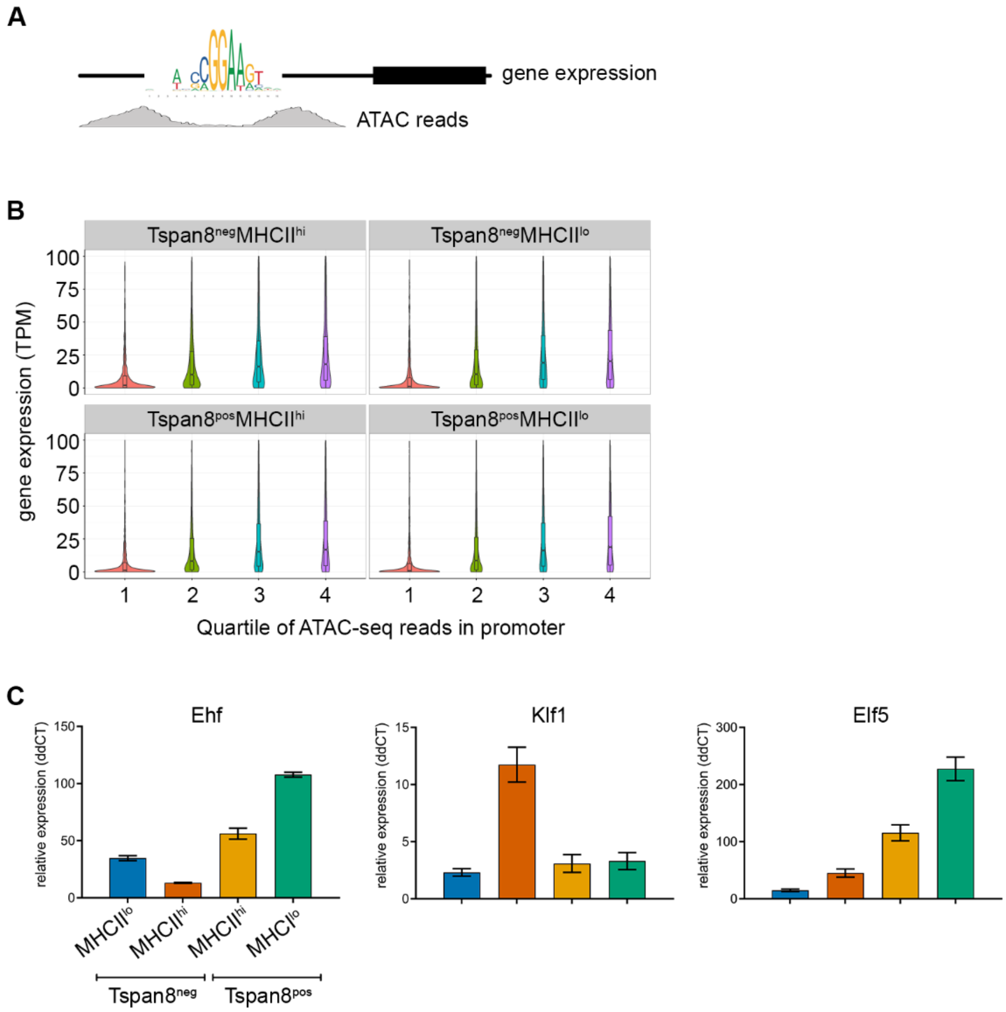

Supplementary Figure 4. Correlation of differential ATAC-seq reads in Tspan8^{pos} compared to Tspan8^{neg} mTEC subpopulations with target gene expression levels in RNA-seq. (**A**) Schematic representation of the experimental setup. A combinatorial enrichment analysis (Tspan8^{pos} vs. Tspan8^{neg}) was performed for ATAC footprint signals and their correlation with the target gene expression level. (**B**) Expression of genes categorized into quartiles of ATAC sequencing signal in promotor regions. Q1 indicating the 25 % lowest ATAC reads in promotor regions of expressed genes, Q2 indicating the next 25 % lowest, Q3 indicating the second highest ATAC-seq reads in promotor regions and Q4 indicating the 25 % highest ATAC-seq reads in promoter regions of expressed genes. (**C**) Expression values of the transcription factors Ehf, Klf1 and Elf5 in Tspan8^{neg}MHCII^{lo} (blue), Tspan8^{neg}MHCII^{hi} (red), Tspan8^{pos}MHCII^{lo} (green) and Tspan8^{pos}MHCII^{hi} (orange) mTEC populations. Shown are the relative expression values obtained by quantitative real-time PCR, normalized to Actin and total thymus lysate (δδCT method), as mean ± SEM.

A

	ChIP seq
gene	maxFC
Kctd15	9.2
Asxl3	8.2
Prokr2	8.0
Ckmt1	7.3
Kif26a	7.1
Fabp9	5.1
Nol4	4.9
Lyplal1	4.6
Tlcd2	4.5
Pxdn	4.5
Olfm3	4.1
Bbox1	4.0
Resp18	3.5
Col19a1	3.5
Timd2	3.3
Gls2	3.2
Apba1	3.1
Krt10	3.0

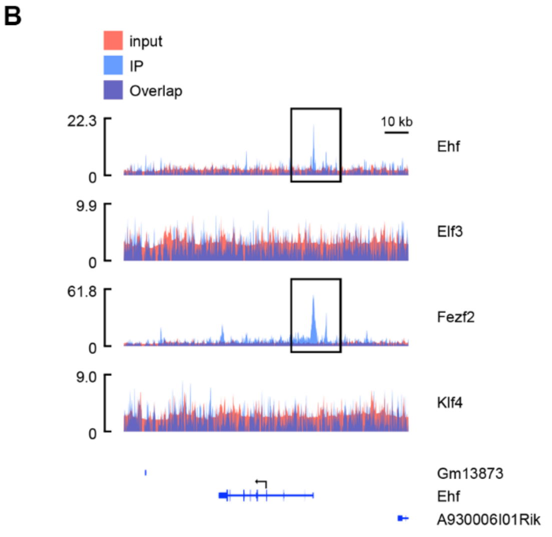

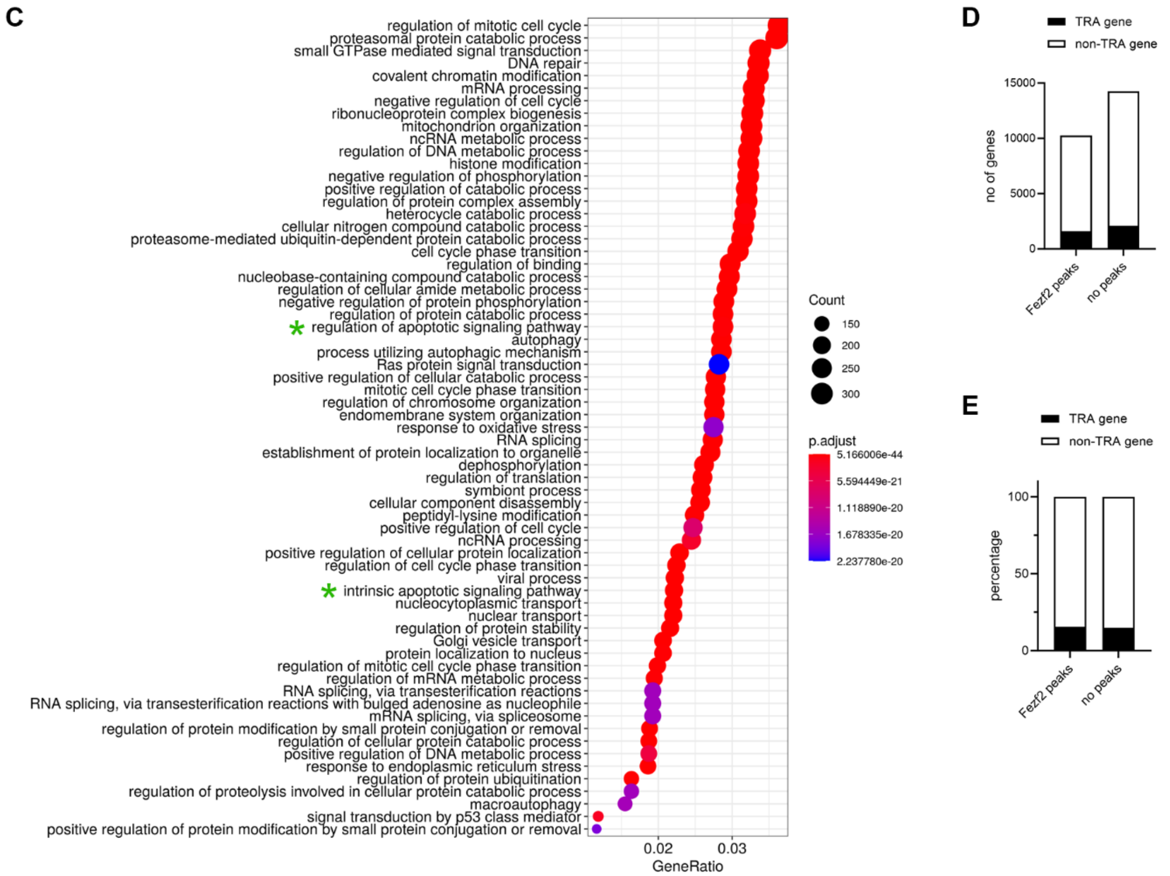

Supplementary Figure 5. Annotation of Fezf2 ChIP-seq target genes. (**A**) Fezf2-dependent genes from the Takaba et. al study (2015) were analyzed for the presence of peaks in our Fezf2 ChIPmentation sequencing dataset. Gene names and the maxFC enrichment in the ChIP compared to the input control are listed. For each gene, maxFC represents the maximum value for the signal enrichment among all peaks within +/- 5000bp from their TSS. (**B**) Signal density plots indicating the read density around the TSS of Ehf. ChIP (blue) and input control (red) tracks for the transcription

factors Ehf, Elf3, Fezf2 and Klf4 are shown. The Y-axis represents the λ score from MACS2, i.e. Read length (nt) * Total read number/ Effective genome length (nt). (**C**) Dotplot showing the 65 most enriched Biological Process GO terms associated with genes that have one or more peaks within +/- 5000 bp of their TSS. The size of the circles indicates the number of genes per term, while colors indicate the significance based on adjusted pvalue using a hypergeometric test. The GeneRatio represents (number of genes related to GO term) / (total number of genes in the query. Apoptosis related annotations are highlighted with the * symbol. (**D**) Number of TRA genes and non-TRA genes with and without Fezf2 ChIP peaks within +/- 5000bp from their TSS. (**E**) Percentage of TRA genes and non-TRA genes with and without Fezf2 ChIP peaks within +/- 5000bp from their TSS.

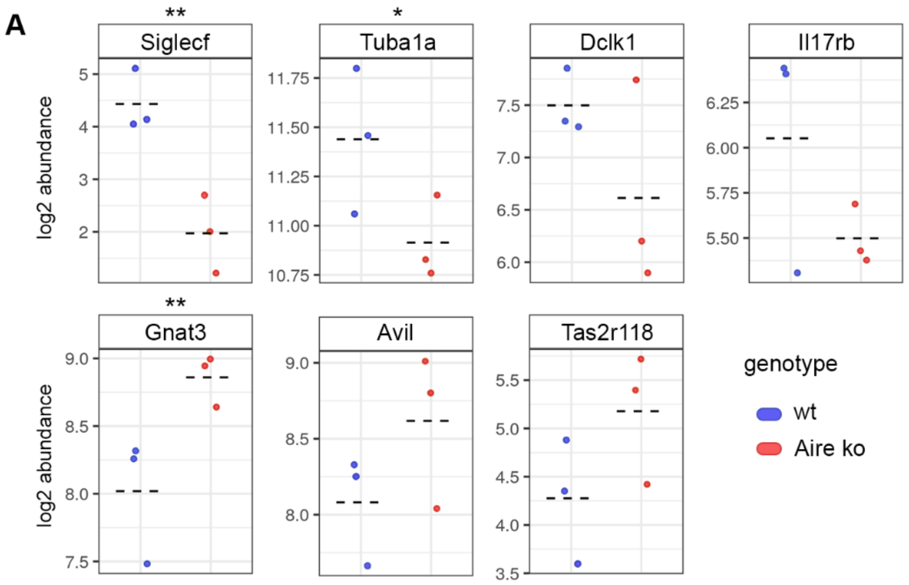

B

Fezf2 ko vs wt

gene name	log2FC	absolute FC	pvalue	padj
Tas2r118	0.5	1.4	0.03	0.24
Siglecf	0.4	1.3	0.07	0.40
Reep5	0.4	1.3	0.06	0.36
Gnat3	0.4	1.3	0.08	0.43
Tas1r1	0.3	1.2	0.14	0.5
Tuba1a	0.16	1.1	0.4	0.8
Tas1r3	0.06	1.0	0.7	0.9
Tas2r138	0.03	1.0	0.8	1.0
Tas2r134	0.01	1.0	0.9	
Pde2a	0.00	1.0	1.0	1.0
Plcg2	-0.02	1.0	0.9	1.0
Gfi1b	-0.02	1.0	0.9	1.0
Tas1r2	-0.04	1.0	0.6	
Chat	-0.08	1.1	0.6	0.9
Il25	-0.15	1.1	0.3	0.7
Plcb2	-0.21	1.2	0.3	0.7
Avil	-0.26	1.2	0.2	0.6
Il17rb	-0.27	1.2	0.2	0.6
Dclk1	-0.30	1.2	0.1	0.5
Trpm5	-0.30	1.2	0.1	0.5
Ptgs1	-0.4	1.3	0.09	0.4
Pou2f3	-0.6	1.5	0.02	0.18
Alox5	-0.7	1.6	0.01	0.12
Alox5ap	-1.0	2.0	0.01	0.10
Rgs13	-1.0	2.0	0.01	0.09
Ltc4s	-1.1	2.1	0.002	0.05

Aire ko vs wt

gene name	log2FC	absolute FC	pvalue	padj
Gnat3	0.7	1.6	0.002	0.03
Avil	0.4	1.3	0.06	0.27
Tas2r118	0.3	1.3	0.1	0.41
Pde2a	0.25	1.2	0.2	0.6
Rgs13	0.25	1.2	0.2	0.6
Plcb2	0.24	1.2	0.2	0.6
Tas1r1	0.22	1.2	0.2	0.6
Il25	0.20	1.1	0.1	0.5
Pou2f3	0.20	1.1	0.3	0.7
Alox5ap	0.20	1.1	0.3	0.6
Tas1r3	0.14	1.1	0.5	0.8
Trpm5	0.08	1.1	0.6	0.9
Alox5	0.06	1.0	0.7	0.9
Reep5	0.05	1.0	0.7	0.9
Tas2r134	0.04	1.0	0.5	
Tas2r138	0.02	1.0	0.9	1.0
Ptgs1	-0.01	1.0	1.0	1.0
Tas1r2	-0.03	1.0	0.4	
Gfi1b	-0.06	1.0	0.7	0.9
Ltc4s	-0.12	1.1	0.5	0.8
Chat	-0.13	1.1	0.5	0.8
Plcg2	-0.23	1.2	0.18	0.5
Il17rb	-0.32	1.3	0.14	0.45
Dclk1	-0.33	1.3	0.13	0.44
Tuba1a	-0.5	1.4	0.01	0.11
Siglecf	-1.8	3.4	0.003	0.03

padj ≤ 0.5

padj ≤ 0.1

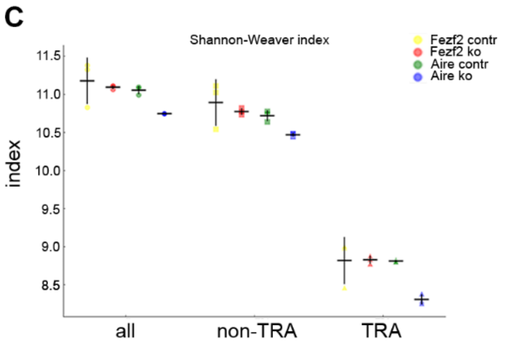

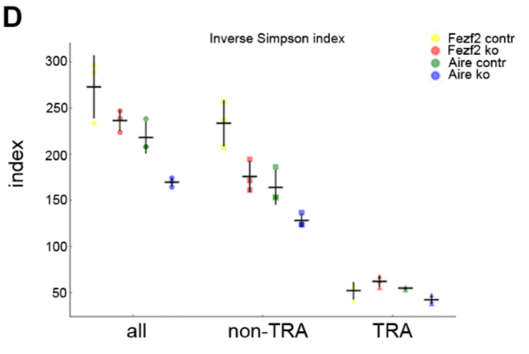

Supplementary Figure 6. Fezf2 and Aire differently impact Tuft cell gene signature expression in mTECs. (**A**) Gene expression plots indicate the log2 abundance of transcript for Tuft cell signature genes which are differentially expressed in the Aire ko compared to wt based on the RNA-seq dataset by Tomofuji et al.. wt (blue), Aire ko (red), dashed lines indicate the mean; upper panel: down-regulated genes; lower panel: up-regulated genes; **≤0.05; *≤0.1 (**B**) Listed are the analyzed Tuft cell

signature genes and their log2 fold change, absolute fold change, p-value and adjusted p-value for the Fezf2 ko compared to wt (left; orange) and Aire ko compared to wt (right; red) gene expression analysis based on the RNA-seq datasets by Tomofuji et al.. grey scale indicates adjusted p-value ≤ 0.1 and ≤ 0.5. (**C**) Shannon-Weaver index is shown for Fezf2 control (contr), Fezf2 knockout (ko), Aire control (contr) and Aire knockout (ko) RNA-seq samples and the gene populations all genes, non TRA genes and TRA genes. Shown are the mean ± SD. (**D**) Inverse Simpson index is shown for Fezf2 control (contr), Fezf2 knockout (ko), Aire control (contr) and Aire knockout (ko) RNA-seq samples and the gene populations all genes, non TRA genes and TRA genes. Shown are the mean ± SD.

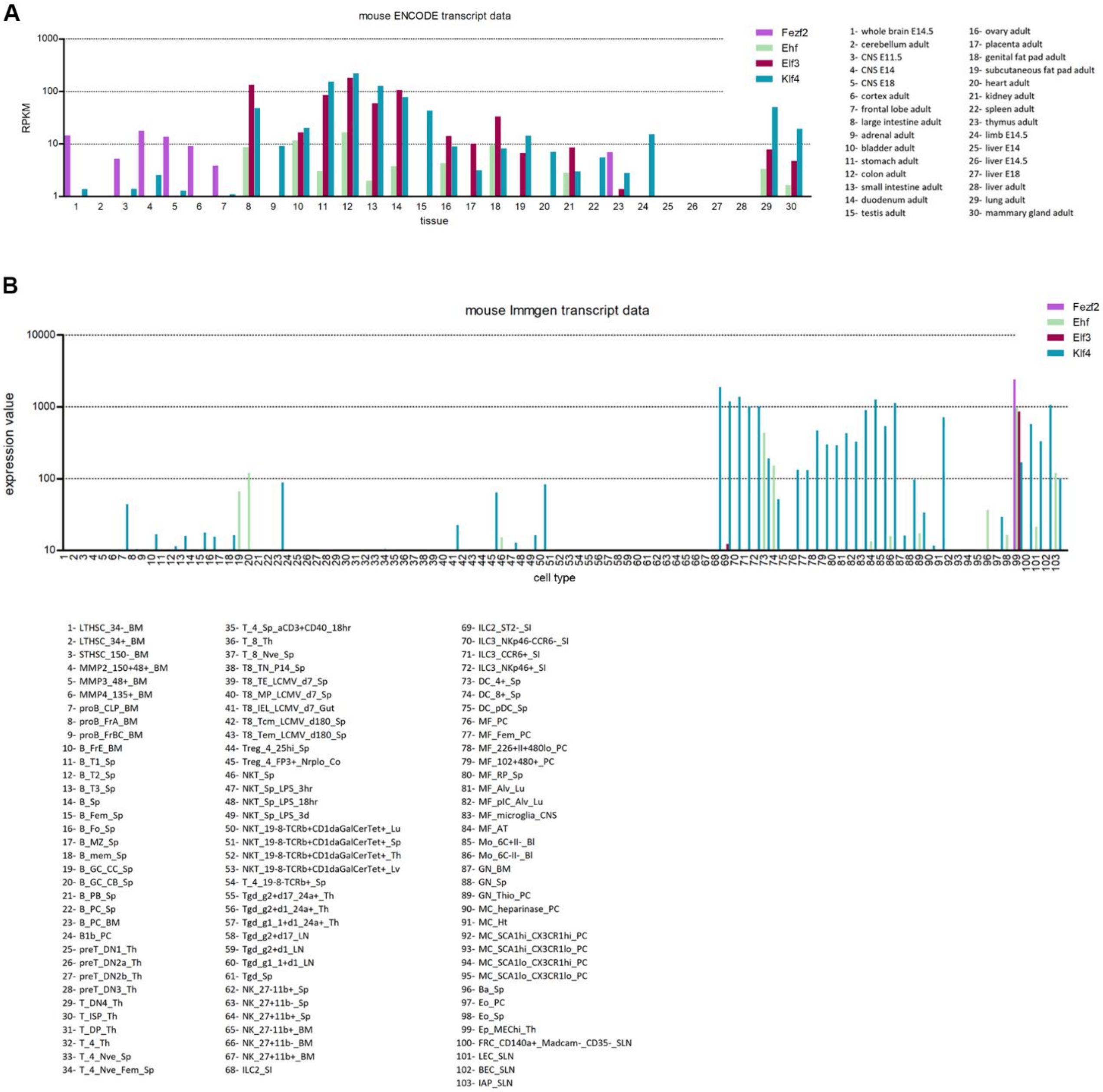

Supplementary Figure 7. Fezf2, Ehf, Elf3 and Klf4 expression profiles in peripheral tissues and immune cell subsets. (**A**) Tissue comparison of gene expression levels (in RPKM; reads per kilobase of transcript) for the transcription factors Fezf2, Ehf, Elf3 and Klf4 based on mouse ENCODE transcript data. (**B**) Immune cell type comparison of gene expression levels (normalized expression values by DESeq2) for the transcription factors Fezf2, Ehf, Elf3 and Klf4 based on the mouse Immgen database. A comprehensive metadata on the cell population names and FACS gating information can be found online at http://rstats.immgen.org/Skyline/skyline.html
